# Cytotoxic Effects of *Citrus* Peels on Breast Tumor: Opportunities for Waste to Raw Material Conversion

**DOI:** 10.1021/acsomega.5c01170

**Published:** 2025-04-17

**Authors:** Ömer Faruk Yakıncı, Esra Emerce, Perihan Gürbüz, Mürşide Ayşe Demi̇rel, Songül Çeri̇başı, İpek Süntar

**Affiliations:** † National Poisons Information Service, Republic of Türkiye Ministry of Health, Ankara 06680, Türkiye; ‡ Institute of Health Sciences, Gazi University, Ankara 06560, Türkiye; § Department of Pharmaceutical Toxicology, Faculty of Pharmacy, Gazi University, Ankara 06630, Türkiye; ∥ Department of Pharmacognosy, Faculty of Pharmacy, Erciyes University, Kayseri 38280, Turkey; ⊥ Department of Pharmaceutical Basic Sciences, Faculty of Pharmacy, Gazi University, Ankara 06630, Türkiye; # Department of Pathology, Faculty of Veterinary Medicine, Fırat University, Elazığ 23119, Türkiye; ¶ Department of Pharmacognosy, Faculty of Pharmacy, Gazi University, Ankara 06630,Türkiye

## Abstract

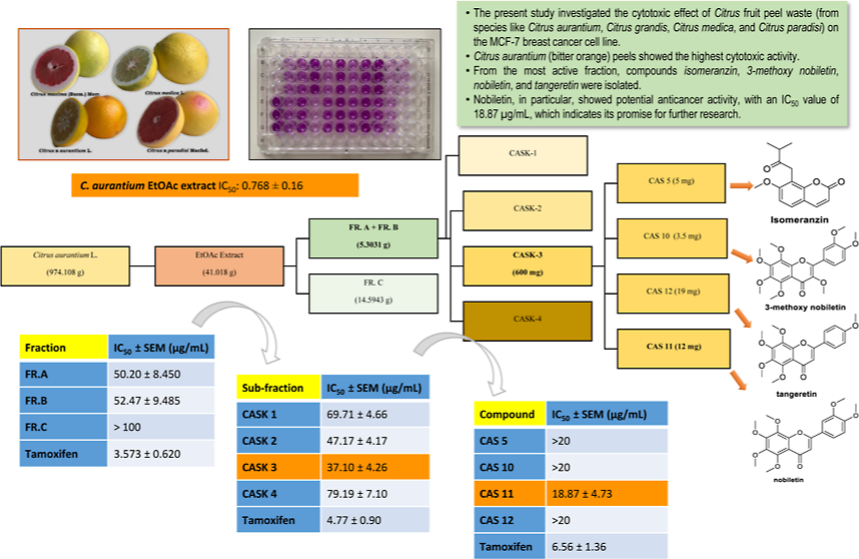

*Citrus* species have long been known for their rich nutritional value. Recent research has shed light on their therapeutic potential, particularly in cancer treatment. *Citrus* peels, on the other hand, often discarded as waste, contain a wealth of bioactive compounds, such as flavonoids, coumarins, and essential oil components, which have proven medicinal properties. Converting *Citrus* peels from waste products to medicinal raw materials is a crucial approach in both healthcare and sustainability. Therefore, the present study aims to investigate the cytotoxic potential of the peels of *Citrus aurantium* L., *Citrus maxima* (Burm.) Merr. (syn. *Citrus grandis*), *Citrus medica* L. and *Citrus paradisi* Macfad. cultivated in Türkiye, and to find out the compounds responsible for the cytotoxic activity. The cytotoxic effects of the peel extracts were evaluated on MCF-7 cell lines according to bioactivity-guided fractionation and isolation assay procedures. The compounds **CAS-5** (isomeranzin), **CAS-10** (3-methoxy nobiletin), **CAS-11** (nobiletin), and **CAS-12** (tangeretin) were isolated. *In silico* analyses conducted on the isolated compounds provided supporting information for the results obtained from *in vitro* experiments regarding their anticancer activity. Indeed, one of the key components of *Citrus* fruits is polymethoxy flavonoids (PMFs), a group of bioactive constituents recognized for their anti-inflammatory, antioxidant, and anticancer activities. As a valuable byproduct of *Citrus* waste, PMFs offer a dual benefit by reducing waste while providing a natural source of bioactive compounds and making them an exciting research area in cancer management. The therapeutic promise of PMFs lies not only in their ability to combat cancer but also in their potential to contribute to sustainable practices.

## 1 Introduction

*Citrus* fruits are a vital agricultural product that greatly boosts the economies of nations producing them. According to the FAO, about 130 million tons of *Citrus* fruits are produced worldwide each year, making them the third-largest fruit crop in the world after apples and bananas, according to the FAO.^1^ Particularly, oranges, lemons, limes, and grapefruits are so widely consumed fruits; therefore, a significant amount of *Citrus* peel waste is created annually throughout the world. Approximately 50–60% of peel waste is produced by the *Citrus* processing industry. Worldwide, the *Citrus* processing industry generates more than 60 million tons of waste.^2^*Citrus* peel needs to be managed and used in a more sustainable manner, as this chart illustrates its significant amount. Potential applications for *Citrus* waste include the production of jams, marmalades, flavorings, dietary supplements, animal feed, biosorbents, bioplastics, biofertilizers, and biogas. Accordingly, investigating the potential applications of natural resources in cancer research, including their effects on breast tumors, is of significant scientific and clinical interest. *Citrus* species are medicinal and aromatic plants with antitumor, cardioprotective, antioxidant, bactericidal, and antiviral properties. Thanks to these beneficial effects, they have potential applications as nutraceuticals, traditional herbal medicinal products, and herbal medicines.^3^ Epidemiological studies suggest that *Citrus* fruits may play a role in the prevention of various types of cancer.^4^ These species, which contain bioactive compounds with diverse chemical structures such as flavonoids, coumarins, and limonoids, have been shown to exert positive effects on inflammation and oxidative processes, thus potentially reducing the risk of diabetes, cardiovascular diseases, and cancer.^5^

In 2022, around 2.3 million new cases of breast cancer were reported worldwide, and the disease was the cause of roughly 665.000 deaths.^6^ This highlights the critical importance of advancing breast cancer treatment options, encompassing *in vitro*, *in silico*, and *in vivo* approaches. Cell lines are extensively utilized in various aspects of laboratory research, particularly as *in vitro* models in cancer studies, making them a critical tool for the molecular investigation of breast cancer. Among these, MCF-7 cells are particularly noteworthy, as they are widely employed in research involving estrogen receptor (ER)-positive breast cancer. Furthermore, the numerous established subclones of MCF-7 cells represent diverse classes of ER-positive tumors exhibiting varying levels of nuclear receptor expression.^7^ Exploring novel compounds with anticancer properties remains a vital area of research. Notably, over 50% of anticancer drugs approved by the U.S. Food and Drug Administration (FDA) are derived from natural sources.^8^

By taking the mentioned information into account, and due to their potential active compound groups, we aimed to perform *in vitro* investigations on the peels of *Citrus aurantium* L. (bitter orange), *Citrus maxima* (Burm.) Merr. (pomelo), *Citrus medica* L. (citron), and *Citrus paradisi* Macfad. (grapefruit) on the MCF-7 ER-positive breast cancer cell line by using the MTT assay. The objective was to isolate and characterize the active compounds by *in vitro* cytotoxic activity-guided fractionation and isolation assays. Moreover, we revealed the compounds’ anticancer activities through *in silico* methods by analyzing their structure–activity relationships.

## 2 Materials and Methods

### 2.1 Plant Material

**Figure 1:**
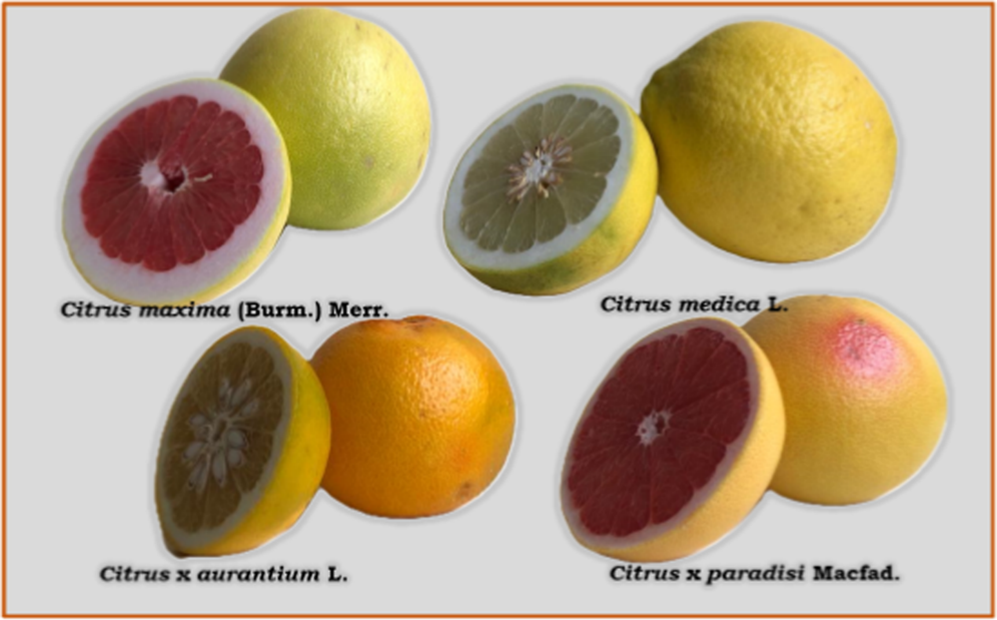
Fruits of *Citrus* sp. (photographed by Ö.F. YAKINCI).

The fruits of *C. aurantium* L., *C. maxima* (Burm.) Merr. (syn. *Citrus grandis*), *C. medica* L. and *C. paradisi* Macfad. were obtained from the Batı Akdeniz Agricultural Research Institute (Antalya, Meditteranean Region), Türkiye, in January 2021 (Figure 1). Species identifications were made by Agricultural Engineer Dr. Ertuğrul TURGUTOĞLU from the Horticulture Department of Batı Akdeniz Agricultural Research Institute. Herbarium samples of the plants were registered at the Gazi University Faculty of Pharmacy Herbarium (GUEF) with the numbers GUEF 3837, GUEF 3838, GUEF 3839, and GUEF 3840, respectively. Since the abbreviated names of the species are used in the present study [*C. aurantium* (CA), *C. grandis* (CG), *C. medica* (CM), and *C. paradisi* (CP)], *C. grandis*, a synonym of *C. maxima* was used to avoid confusion with the abbreviation of *C. medica* (CM).

### 2.2 Extraction of the Fruit Peels of *Citrus* sp

The peels of the fruits were separated, dried in the shade, and ground into powder for maceration. Each sample (10 g) was directly macerated with ethyl acetate (EtOAc, 200 mL) at room temperature for 24 h using a shaker, repeated three times. The resulting extracts were filtered, and the solvent was removed under reduced pressure and low temperature (40 °C) using a rotary evaporator (Buchi Rotavapor R-100, Flawil, Switzerland). This process yielded four crude EtOAc extracts corresponding to the four species. The extraction yields were calculated as 0.667%, 0.180%, 0.728%, and 1.286%, respectively.

### 2.3 Extraction, Fractionation and Isolation Studies on the Fruit Peels of *C. aurantium*

The air-dried peels of *C. aurantium* (974.108 g) were extracted (macerated) with EtOAc for 24 h at room temperature (3 × 5 L), and then, the combined extracts were evaporated under reduced pressure and low temperature (40 °C) to give crude extracts (EtOAc: 41.018, 4.21% yield). A portion of EtOAc extract (23 g) was first subjected to Sephadex LH-20 (SP LH-20) column chromatography (5 × 100 cm) and eluted with CH_2_Cl_2_:MeOH (1:1) to give three main fractions (SP FR. A.: 2.6815 g, SP FR. B.:2.6216, SP FR. C.:14.5943 g). FR. A and FR. B were combined according to similar activity results and thin layer chromatography (TLC) profiles before further chromatographic steps to increase the amount of active compound at the final purification step.

FR.A + B (5.3 g) was first subjected to silica gel column (4 × 100 cm) and eluted with increasing polarity of *n*-hexane: EtOAc mixtures [(8:2; 800 mL v/v), (1:1; 1000 mL, v/v), (2:8; 500 mL v/v)], EtOAc (500 mL), EtOAc: MeOH mixtures [(8:2; 500 mL, v/v), (1:1; 500 mL, v/v)] and MeOH (500 mL) to give four subfractions [CASK-1:1.3 g; CASK-2:730 mg; CASK-3:600 mg, CASK-4:1.4 g].

CASK-3 (600 mg) was chromatographed over a prepacked silica gel column (80 g) and eluted with *n*-hexane: EtOAc (v/v, 7:3; 400 mL), *n*-hexane: EtOAc (v/v, 5:5; 800 mL), *n*-hexane: EtOAc (v/v, 3:7; 400 mL) respectively to yield seven subfractions (Fr a-g). Fr a (8 mg) was further purified with SP LH-20 column chromatography (1.5 × 40 cm) and eluted with CH_2_Cl_2_:MeOH (1:1) to give **CAS-5** (5 mg). Fr c (20 mg) was eluted with MeOH through SP LH-20 to give compound **CAS-10** (3.5 mg). Fr d (40 mg) and Fr g (70 mg) were separately dissolved in MeOH: *n*-hexane mixtures and left overnight for spontaneous crystallization at room temperature. Then, needles were washed with Et_2_O and C_6_H_6_ to yield pure compounds **CAS-12** (19 mg) and **CAS-11** (12 mg), respectively. The isolated compounds were characterized using spectroscopic techniques (1D, 2D NMR, and MS).

### 2.4 *In Vitro* Cytotoxicity Evaluation

The MTT assay was employed to determine the cytotoxic effect of the samples on breast cancer cells. MCF-7, a human estrogen receptor-positive breast cancer cell line, was used to evaluate cytotoxicity, and MCF-10A, a healthy human breast cell line, to calculate the selectivity index (SI).^9^

#### 2.4.1 Cell Culture Conditions

MCF-7 cells (HTB-22, ATCC) were cultured in DMEM medium supplemented with 10% FBS, 1% l-glutamine, and 1% antibiotics (penicillin–streptomycin) at 37 °C in a humidified incubator with 5% CO_2_. MCF-10A cells (CRL-10317, ATCC) were cultured in DMEM/Ham’s F12 medium supplemented with 20 ng/mL epidermal growth factor, 500 ng/mL hydrocortisone, 10 μg/mL insulin, 10% FBS, 100 U/mL penicillin, and 100 μg/mL streptomycin under the same incubation conditions.

#### 2.4.2 MTT Assay

Cell counting was performed using the trypan blue exclusion method,^10^ and 5000 cells/well were seeded into 96-well plates. Extracts, fractions, subfractions, or isolated compounds were applied at increasing concentrations according to the experimental design. Dose selection was based on the National Cancer Institute (NCI) anticancer drug screening program, with the maximum concentration for plant extracts and fractions set at 100 μg/mL.^11^ For isolated compounds, the maximum concentration was set at 20 μg/mL, considering that the IC_50_ value of promising anticancer agents should be ≤4 μg/mL.^12^ The experiment also included negative controls, solvent controls (DMSO at a final concentration of 0.1%), and positive controls (tamoxifen). After 48 h of treatment, the cells were washed, fresh medium and MTT solution (1 g/L) were added to the wells, and the cells were incubated for 3 h. Subsequently, the mixture was removed, DMSO was added, and absorbance values were measured at 570 nm using a plate reader. The cytotoxic activity assay was performed three times for each sample, with at least duplicate wells for each experiment (*n* = 6), and IC_50_ values were calculated.

### 2.5 *In Silico* Biological Activity Assessments

The isolated and structurally elucidated compounds were analyzed for their anticancer activities through computer-based biological activity evaluation. For this purpose, the PASS (Prediction of Activity Spectra for Substances) program was utilized.^13^ The probabilities of being active (Pa) for relevant biological activities associated with anticancer effects were determined. Additionally, the potential cytotoxic effects of the compounds on cancer cell lines were predicted using the CLC-Pred (Cell Line Cytotoxicity Predictor) software (v. 2.0).^14^ To assess mutagenic potential, evaluations were conducted using the VEGA QSAR Mutagenicity Consensus Model (v. 1.0.4),^15^ ToxTree (SAR) Mutagenicity Model (v. 3.1.0),^16^ and ToxRead (0.25 Beta) (Read-across)^17^ approaches.

### 2.6 Statistical Analysis

Statistical data analysis was performed using GraphPad Prism (GraphPad Prism Software, v.8, USA). The results were calculated as mean ± standard error of the mean (SEM). The IC_50_ values were calculated from nonlinear regression analysis.

## 3 Results

### 3.1 Phytochemical Analysis

**Figure 2:**
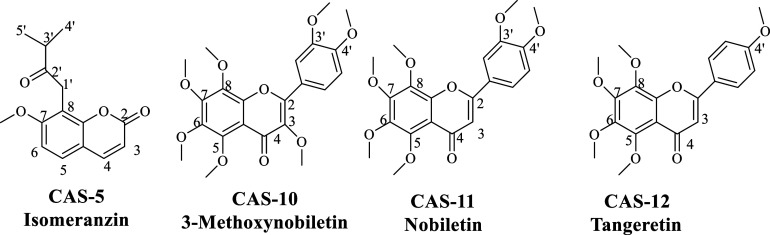
Structures of isolated compounds from *Citrus aurantium*.

Repeated chromatographic experiments resulted in the separation of one coumarin and three methoxylated flavonoids via bioactivity-guided approach from ethyl acetate (EtOAc) extract (Figure 2). The structures of the compounds were elucidated using 1D (^1^H, ^13^C) and 2D (HSQC, HMBC) NMR analyses in conjunction with MS data and comparison with relevant literature. Substances were identified and described as isomeranzin (**CAS-5**)^3,18^-methoxynobiletin (**CAS-10**),^19^ nobiletin (**CAS-11**)^20^ and tangeretin (**CAS-12**).^21^

### 3.2 *In Vitro* Cytotoxicity Tests

Based on the obtained findings and considering the activity results, it was determined that the CA EtOAc crude extract exhibited the highest cytotoxic effect (IC_50_: 0.77 ± 0.16 μg/mL) (Table 1). Therefore, to assess the potential damage the CA EtOAc crude extract could cause to healthy breast cells, the same extract was also applied to the MCF-10A cell line. The study revealed that the IC_50_ value of the CA EtOAc crude extract in the MCF-10A healthy breast cell line was 30.07 ± 1.62 μg/mL. The selectivity index (SI) was calculated as 39.05, indicating a high selectivity of the extract to the targeted cancer cells.

**Table 1:** Cytotoxic Effects (IC_50_ Values) of *Citrus* EtOAc Extracts on the MCF-7 Cell Line^a^

groups	IC_50_ ± SEM
CA	0.77 ± 0.16 μg/mL
CG	3.34 ± 0.36 μg/mL
CM	1.26 ± 0.17 μg/mL
CP	13.41 ± 1.59 μg/mL
tamoxifen	2.18 ± 0.37 μM

a CA: *Citrus aurantium*; CG: *Citrus grandis*; CM: *Citrus medica*; CP: *Citrus paradisi*.

Fractionation studies were continued with CA EtOAc extract due to its strong cytotoxic effect. SP FR.A, SP FR.B, and SP FR.C fractions of CA EtOAc extract, obtained from the initial SP LH-20 column, were applied to MCF-7 and MCF-10A cells at doses of 100, 31.6, 10, 3.2, and 1 μg/mL. According to the results (Table 2), the IC_50_ values for the SP FR.A and SP FR.B fractions in MCF-7 cells were found to be 50.20 ± 8.45 and 52.47 ± 9.46 μg/mL, respectively. The SP FR.C fraction, however, did not exhibit significant cytotoxic activity even at the highest dose (IC_50_ > 100 μg/mL). When evaluating the cytotoxicity of the CA EtOAc extract fractions from the SP LH-20 column on the MCF-10A cell line, the IC_50_ for SP FR.A was found to be 59.67 ± 9.94 μg/mL, while no cytotoxicity was observed at the tested concentrations for the other fractions. The selectivity indices of the subfractions were calculated to be 1.19 for SP FR.A and >1.91 for SP FR.B.

**Table 2:** Cytotoxic Effects and Selectivity Indices of the SP LH-20 Column Fractions of the CA EtOAc Extract on the MCF-7 and MCF-10A Cell Lines^a^

fraction	IC_50_ ± SEM (MCF-7)	IC_50_ ± SEM (MCF-10A)	Selectivity index
SP FR.A	50.20 ± 8.45 μg/mL	59.67 ± 9.94 μg/mL	1.19
SP FR.B	52.47 ± 9.46 μg/mL	>100 μg/mL	>1.91
SP FR.C	>100 μg/mL	>100 μg/mL	
tamoxifen	3.57 ± 0.62 μM	7.47 ± 1.30 μM	2.09

a SP: Sephadex LH-20; FR: Fraction.

However, the activity observed in MCF-7 cells at a concentration of 100 μg/mL for the SP FR.A and SP FR.B fractions were not detected at lower doses. This suggests that the strong cytotoxic effect observed in the CA EtOAc crude extract (IC_50_: 0.768 ± 0.16 μg/mL) was reduced following fractionation. Given that the effects of the compounds in the crude extract were diminished after fractionation, it is presumed that any potential synergistic effect disappeared. Based on these findings, isolation studies were continued, in an activity-guided manner, on the fraction that exhibited the highest activity among the fractions.

The subfractions (CASK 1–4) obtained from the SP LH-20 column fractions (SP FR.A + SP FR.B) using a silica gel column were applied to MCF-7 and MCF-10A cell lines for 48 h to evaluate their cytotoxic effects on the cells (Table 3). CASK-3 was determined to be the most active fraction with the IC_50_ value of 37.10 ± 4.26 μg/mL. The selectivity index was calculated to be 0.847 for CASK-3.

**Table 3:** Cytotoxic Effects and Selectivity Indices of the Silica Gel Column Fractions (Sub-fractions) on the MCF-7 and MCF-10A Cell Lines

subfraction	IC_50_ ± SEM (MCF-7)	IC_50_ ± SEM (MCF-10A)	selectivity index
CASK-1	69.71 ± 4.66 μg/mL	68.15 ± 3.01 μg/mL	0.977
CASK-2	47.17 ± 4.17 μg/mL	43.30 ± 2.11 μg/mL	0.917
CASK-3	37.10 ± 4.26 μg/mL	31.43 ± 1.44 μg/mL	0.847
CASK-4	79.19 ± 7.10 μg/mL	87.02 ± 11.86 μg/mL	1.099
tamoxifen	4.77 ± 0.90 μM	11.03 ± 0.63 μM	2.312

Cell culture studies were conducted on MCF-7 and MCF-10A cell lines using compounds isolated from CASK-3, including **CAS-5** (isomeranzin), **CAS-10** (3-methoxy nobiletin), **CAS-11** (nobiletin), and **CAS-12** (tangeretin). Among the isolated compounds tested in MCF-7 cells, CAS 11 was identified as the most active, with the IC_50_ value of 18.87 ± 4.73 μg/mL (46.9 ± 11.70 μM). The other compounds did not show significant cytotoxic activity at the tested concentrations (Table 4).

**Table 4:** Cytotoxic Effects and Selectivity Indices of Compounds Isolated from CASK-3 on the MCF-7 and MCF-10A Cell Lines

compound	IC50 ± SEM (MCF-7)	IC50 ± SEM (MCF-10A)	selectivity index
**CAS-5** (isomeranzin)	>20 μg/mL	>20 μg/mL	
**CAS-10** (3-methoxy nobiletin)	>20 μg/mL	>20 μg/mL	
**CAS-11** (nobiletin)	18.87 ± 4.73 μg/mL	>20 μg/mL	>1.1
**CAS-12** (tangeretin)	>20 μg/mL	17.92 ± 1.21 μg/mL	<0.9
tamoxifen	6.56 ± 1.36 μM	8.00 ± 0.73 μM	1.22

### 3.3 *In Silico* Biological Activity Results

The predicted anticancer activity results for the structurally elucidated compounds are shown in Table 5. Regarding the predicted anticancer activity of isomeranzin, the highest probability of 0.691 was calculated for its potential use in antineoplastic applications. Among the four compounds investigated, isomeranzin exhibited the lowest predicted anticancer activity. The other three compounds, however, showed similar results in terms of anticancer activity, with predicted activities for antineoplastic effects, apoptosis agonist activity, and free radical scavenging effects all having probability values above 0.7.

**Table 5:**
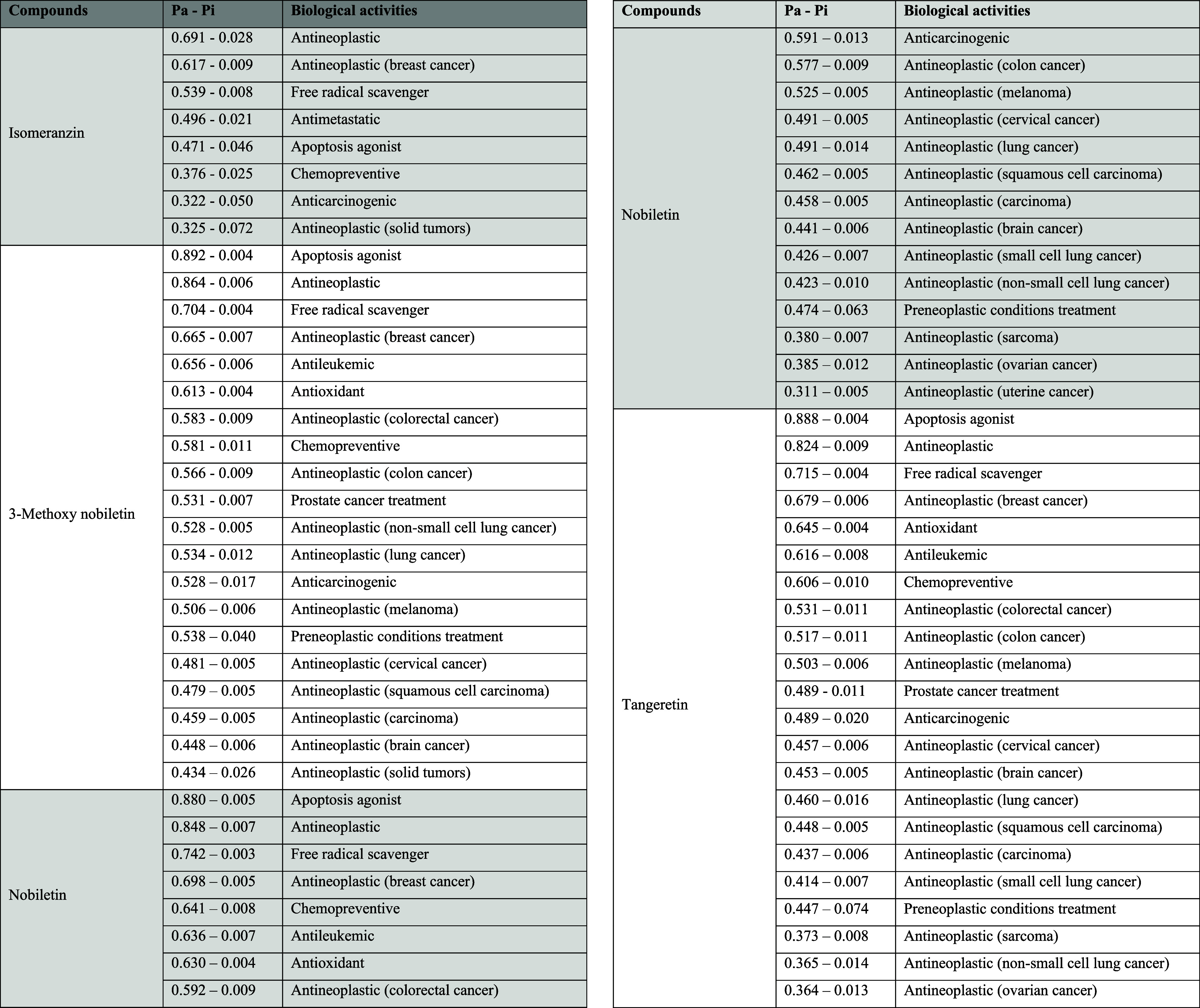
Prediction Results of Isolated Compounds Associated with Anticancer Activity

In the PASS biological activity analysis, independent of anticancer activity, the highest probability activities associated with the compounds, based on the data set, were found as follows: for isomeranzin, cardiovascular analeptic (0.882–0.004), CYP2A11 substrate (0.854–0.003), and CYP2C12 substrate (0.865–0.020). For 3-methoxy nobiletin, the most probable activities were chlordecone reductase inhibitor (0.942–0.003), membrane integrity agonist (0.923–0.003), and HIF1A expression inhibitor (0.910–0.005). For nobiletin, the highest calculated activities were HIF1A expression inhibitor (0.946–0.004), chlordecone reductase inhibitor (0.941–0.003), and membrane integrity agonist (0.919–0.007). Similarly, for tangeretin, the highest predicted activities were chlordecone reductase inhibitor (0.945–0.003), HIF1A expression inhibitor (0.942–0.004), and membrane integrity agonist (0.937–0.004).

The top three cancer cell lines with the highest probability of exhibiting cytotoxic activity, as predicted by CLC-Pred, were analyzed. For isomeranzin, the highest probability (0.646–0.007) was observed for the T47D breast carcinoma cell line, followed by (0.375–0.078) for NALM- adult B acute lymphoblastic leukemia and (0.357–0.060) for HL-60 promyeloblast leukemia cell lines. For 3-methoxy nobiletin, the highest probability of cytotoxic activity was predicted for the CWR22R prostate carcinoma epithelial cell line (0.356–0.009), followed by (0.374–0.054) for HL-60 promyeloblast leukemia and (0.307–0.029) for LS174T colon adenocarcinoma cell lines. For nobiletin, the highest predicted cytotoxic activity was (0.332–0.017) for the CWR22R prostate carcinoma epithelial cell line, (0.361–0.058) for HL-60 promyeloblast leukemia, and (0.323–0.020) for LS174T colon adenocarcinoma cell lines. For tangeretin, the most probable cancer cell lines exhibiting cytotoxic activity were predicted to be HL-60 promyeloblast leukemia (0.377–0.053), CWR22R prostate carcinoma epithelial cells (0.325–0.020), and AGS gastric adenocarcinoma (0.321–0.026).

In the mutagenic activity evaluation (Table 6), the analysis, supported by a cross-referencing approach combining knowledge-based and statistical-based methods, determined that isomeranzin was ″mutagenic.″ In the ToxTree *in vitro* mutagenicity module, the compound was found to belong to the ″coumarins and furocoumarins″ stimulant group. For 3-methoxy nobiletin, although the ToxTree software suggested a structural stimulant related to the flavonoid structure, the overall evaluation concluded that the compound was ″not mutagenic.″ Similarly, both nobiletin and tangeretin were predicted to be ″not mutagenic″ across all approaches.

**Table 6:** Results of *In Silico* Mutagenic Activity Assessment of Isolated Compounds

compounds	ToxTree	Vega QSAR (score)	ToxRead (score)	Overall assessment
isomeranzin	+	+(0.2)	+(0.51)	mutagenic
3-methoxy nobiletin	+	– (0.45)	–(0.95)	nonmutagenic
nobiletin		–(0.5)	–(0.95)	nonmutagenic
tangeretin		–(0.35)	–(0.81)	nonmutagenic

## 4 Discussion

### 4.1 Importance of *Citrus* Species

*Citrus* species are extremely significant in agriculture, because of their widespread production, economic worth, nutritional and therapeutical advantages. The fruits are rich in coumarins, flavonoids, glycosides, essential oil components, limonoids, vitamins B and C, carotenoids, minerals, and dietary fibers. They represent a unique resource for human health and have potential as nutraceuticals, traditional herbal medicinal products, and phytopharmaceuticals due to their antitumor, cardioprotective, free radical scavenging, bactericidal, and antiviral properties.^3,5^ Notably, flavonoids and volatile compounds, responsible for these effects, are present in higher concentrations in *Citrus* fruit peels.^22^ Therefore, additional economic potential is provided by their byproducts, peels, particularly in the food and medicinal sectors. *Citrus* species continue to be vital to the world’s food and agricultural systems as the demand for them rises.

### 4.2 Objective of the Study

In this context, the present study aims to explore the potential cytotoxic effect of *Citrus* fruit peel waste as a high-value-added product. For this purpose, some *Citrus* species which are grown in the Batı Akdeniz Agricultural Research Institute, Türkiye, namely, *C. aurantium* L. (bitter orange) (CA), *C. grandis* (L.) Osbeck (pomelo) (CG), *C. medica* L. (citron) (CM), and *C. paradisi* Macfad. (grapefruit) (CP) were investigated in terms of cytotoxic activity using the MCF-7 cell line, with subsequent isolation of active fractions based on cytotoxic activity.

### 4.3 Extraction and Cytotoxicity Testing

The extraction yield of the crude ethyl acetate (EtOAc) extract from the peels of *C. aurantium* was found to be relatively low, with a yield of 4.21%. Among the factors that might significantly impact the yield of the extracts are the extraction method and the specific conditions used during the extraction process. Modern extraction techniques like ultrasound or microwave-assisted methods, optimizing extraction parameters, repeating extractions, or modifying solvent mixtures can all help increase the efficiency of extracting polymethoxy flavonoids (PMFs) and other valuable compounds.

In the *in vitro* studies, the highest cytotoxic activity in the MCF-7 cell line was provided by the CA EtOAc crude extract. Consequently, the effect of this extract on the healthy MCF-10A cell line was evaluated, yielding a selectivity index of 39.05. This value indicated that the extract possesses a notably good selectivity. Based on these findings, fractionation studies were initiated on the CA EtOAc extract. Using SP LH-20 column chromatography, three fractions (SP FR.A, SP FR.B, and SP FR.C) were obtained, and then, cell culture studies were performed on these fractions. Cytotoxic activity was assessed via MTT assay after 48 h treatment with MCF-7 cells. Among the fractions, SP FR.A and SP FR.B demonstrated cytotoxic activity, while SP FR.C showed lower activity compared to the other two fractions. Although the most active fractions (SP FR.A and SP FR.B) exhibited lower effects than the crude extract, isolation work was continued based on the activity-directed approach, focusing on the fraction with the highest relative activity. The decrease in activity upon fractionation may be attributed to the potential synergistic effects of the compounds in the extract. The compounds present in the crude extract may exhibit synergistic activity, meaning they demonstrate efficacy only when combined. During the fractionation of the extract, the separation of compounds may result in fractions containing fewer active compounds, as they become diluted or distributed in varying proportions throughout the fractions. This process may lead to a reduction in overall bioactivity compared to the crude extract. Consequently, the crude extract can be considered suitable for herbal formulations without the need for further isolation efforts. On the other hand, the possible formation of aggregates, potentially caused by the enrichment of phenolic compounds in the lower fractions, could hinder interactions with biological targets, thereby reducing efficacy. Therefore, in consideration of the potential for false-positive activity results due to molecular self-assembly, activity-guided fractionation studies were continued to the final isolation stage with the expectation that the compounds exerting bioactivity would be identified. This approach facilitates the acquisition of more informative results for further *in vivo* studies.

### 4.4 Activity-Guided Compound Isolation

Since cytotoxic activity results were similar for SP FR.A and SP FR.B, these two fractions were combined and further separated into four subfractions (CASK 1–4) using silica gel. Among the subfractions, CASK-3 exhibited the highest cytotoxic activity and was subjected to further chromatographic isolation. The cytotoxic effect of CASK-3 was found to be higher than that of the previously obtained SP FR.A and SP FR.B fractions. From CASK-3, one coumarin (isomeranzin), and three methoxylated flavonoids (3-methoxy nobiletin, nobiletin, and tangeretin) were isolated and their structures were characterized using 1D and 2D NMR methods, with results verified by literature data.^18,19,21^ Previous studies have reported the presence of nobiletin, tangeretin, naringenin, naringin, hesperetin, hesperidin, and neohesperidin in the high performance liquid chromatography (HPLC) analysis of EtOAc extracts from *Citrus* fruits.^23^ Additionally, the aqueous alcoholic extracts of *C. aurantium* fruit peels have been found to contain naringin, hesperidin, neohesperidin, diosmin, morin, caffeic acid, chlorogenic acid, coumaric acid, and ellagic acid,^24^ while aqueous alcoholic extracts of the fruits identified neohesperidin, synephrine, and naringin.^25^ Moreover, cold-pressed essential oil from *C. aurantium* revealed coumarin (meranzin, meranzin hydrate, isomeranzin, bergapten, epoxybergamottin hydrate, and osthol) and PMF derivatives (nobiletin, 3-methoxy nobiletin, and tangeretin).^26^

The presence of aglycones in the fruit peel was notable.^27^ In the current study, the compounds isolated from the EtOAc extract of *C. aurantium* fruit peels, including isomeranzin, 3-methoxy nobiletin, nobiletin, and tangeretin, were consistent with those previously reported in various *Citrus* fruit solvent extracts. Furthermore, other *Citrus* species evaluated in the present study (*C. grandis*, *C. medica*, and *C. paradisi*) also exhibited cytotoxic effects in *in vitro* assays. Literature records confirm that these species also contain nobiletin. *Citrus* species besides, *C. aurantium*, *C. grandis*, *C. paradisi*, *C. medica*, that also contain nobiletin were reported as *Citrus reticulate*, *Citrus sinesis*, *Citrus miaray*, *Citrus depressa*, *Citrus mandalina*, *Citrus unshiu*, *Citrus indica*, *Citrus reshni*, *Citrus tachibana*, *Citrus leiocarpa*, *Citrus tardiva*, *Citrus succosa*, *Citrus kinokuni*, *Citrus erythrosa*, *Citrus sunki*, and *Citrus deliciosa* in the previous studies.^20,21,28−34^

Given that an IC_50_ value ≤4 μg/mL is considered promising for anticancer compounds,^12^ the highest concentration tested for the isolated compounds was 20 μg/mL. It was found that isomeranzin, 3-methoxy nobiletin, and tangeretin did not show significant effects against MCF-7 cells at this concentration. Nevertheless, nobiletin exhibited promising activity, with an IC_50_ value of 18.87 ± 4.73 μg/mL, indicating potential for further research and development.

The IC_50_ values of isomeranzin, a coumarin derivative, and 3-methoxy nobiletin and tangeretin, which are flavonoid compounds, remained above 20 μg/mL in MCF-7 cell lines. In cytotoxicity assays performed on MCF-10A healthy cells with the isolated compounds, it was determined that nobiletin exhibited a selectivity index (SI) greater than “1”. Based on these findings, nobiletin emerged as a promising candidate for further evaluation studies. In the present study, based on *in vitro* activity-guided isolation, nobiletin was determined to be largely responsible for the activity observed from *C. aurantium*. However, the diminishing activity profile upon fractionation of the extract suggests that other secondary metabolites of the extract may also contribute to its biological activity.

The current study focuses on four *Citrus* species cultivated in Türkiye, whose fruit peels are generally considered waste material despite the fruits being used as food. Notably, no prior research has investigated the cytotoxic activity of the peels of selected species on the MCF-7 cell line. The primary objective of this study is to determine which species is superior in terms of the conversion of a pharmaceutical raw material. The cytotoxic activity of key compounds found in *Citrus* species (nobiletin, tangeretin, and hesperetin) has been previously demonstrated in earlier studies,^35^ forming the conceptual hypothesis of our research. Through an activity-guided fractionation and isolation approach, the present study successfully identified the bioactive compounds, and the obtained results were consistent with findings from prior research. Future *in vivo* studies will focus on investigating the most effective extract, subextract, fraction, subfraction, and compound group in biological processes.

### 4.5 Comparative Analysis with Other *Citrus* Species

An *in vitro* study reported that flavonoids from *C. aurantium* exhibited antitumor activity by inducing cell cycle arrest in the G2/M phase and apoptosis, decreasing the expression of cdc2, cdc25c, and cyclin B1, and increasing the expression of p21WAF1/CIP1.^36^ Cytotoxicity studies on *C. aurantium* compounds have primarily focused on nobiletin and tangeretin and their derivatives, which have demonstrated cytotoxic effects, particularly against breast, prostate, colon, and colorectal cancer cell lines.^26,35,37−44^ Tangeretin and nobiletin have been reported to inhibit the development of JCS leukemia cells.^45^ Nobiletin was shown to have cytotoxic effects in the MCF-7 cell line in the previous studies.^43,46,47^

A recent study by Wu et al. (2023) demonstrated that nobiletin exhibited an IC_50_ value of 121.78 μM on MCF-7 cells after 72 h of treatment, inhibiting invasion and migration in both T47D and MCF-7 cell lines, suppressing breast cancer progression, and preventing liver metastasis.^48^ Additionally, Chen et al. (2014) reported that nobiletin displayed cytotoxic activity on the MCF-7 cell line, with IC_50_ values of 59.8, 39.7, and 36.6 μM after 72, 120, and 168 h of treatment, respectively.^37^ These values align with our findings. On the other hand, tangeretin has been reported to exhibit cytotoxic activity in MDA-MB-468 cells, while demonstrating lower activity in MCF7 cells,^49^ suggesting a potential estrogen-independent mechanism. Additional mechanistic studies on various cell lines have shown that tangeretin induces apoptosis in human gastric cancer AGS cells through p53-dependent mitochondrial dysfunction and the Fas/FasL-mediated extrinsic pathway.^50^ Furthermore, it has been found to inhibit the Stat3 signaling pathway, contributing to cancer stem cell death. The studies aimed at preparing derivatives of this compound to enhance cytotoxic activity.^40^

### 4.6 Flavonoids and Their Mechanisms

Flavonoids show a distinct mode of action by modulating many metabolic pathways to produce their anticancer effects, in contrast to molecular inhibitors and conventional chemotherapeutics, which primarily target a single metabolic pathway. Particularly, PMFs have recently been shown to have potential anticancer properties, especially through the activation of cell cycle arrest, inhibition of tumor-invasive activity, and regulation of reactive oxygen species on cancer cells.^51−53^ Their synergistic cytotoxic effects have been investigated in recent research, especially in combination with conventional chemotherapeutic drugs. In resistant cancer cell lines, tangeretin has been demonstrated to increase paclitaxel’s cytotoxicity. Increased apoptosis and cell cycle arrest in the G2/M phase were the results of the combination, indicating a potential synergistic effect. In MX-1 and MX-1/T cells, sinensetin has been shown to decrease cell viability and enhance cytotoxic effect of paclitaxel, suggesting that it may be able to overcome drug resistance.^54^ Furthermore, Ma et al. (2015) demonstrated that nobiletin significantly sensitized ABCB1-overexpressing A2780/T and A549/T cells to chemotherapeutic agents, including paclitaxel, doxorubicin, docetaxel, and daunorubicin. Based on these findings, they proposed that nobiletin, as part of combination therapy, could be a promising candidate for *in vivo* studies aimed at reversing ABCB1-mediated drug resistance in cancer treatment.^55^ The synergistic cytotoxicity seen with PMFs and chemotherapeutic combinations presents a viable way to improve the effectiveness of cancer treatment. These interactions not only increase the effectiveness of currently available medications but also offer ways to fight drug resistance, which could result in more individualized and efficient cancer treatments. However, it is essential to comprehend the long-term effects of flavonoid-based interactions with certain metabolic targets in order to assess their safety and effectiveness in the treatment of cancer.^56,57^ Other important consideration for therapeutic compounds is their bioavailability. The compound’s solubility and permeability across physiological barriers suggest proper absorption. It has been demonstrated that nobiletin has low oral bioavailability (<1%) and low water solubility (1–5 μg/mL), which reduces its biological and therapeutic activities.^58^ Numerous approaches have been studied to improve the bioavailability of nobiletin, including the use of ionic liquids, encapsulation strategies, and nanoemulsion techniques.^59^

### 4.7 *In Silico* Study

The aim of the *in silico* study is to predict anticancer activity outcomes for four compounds, which were isolated through activity-guided fractionation, and to provide Supporting Information for the results obtained from existing *in vitro* experiments. Initially, a probabilistic evaluation of anticancer activity is conducted through computational-based biological activity assessment. Additionally, based on the existing databases for biological activity, the three most likely activities associated with each compound are predicted. However, when interpreting the results, it is important to note that if a compound is predicted to have a low probability of activity, this may indicate the absence of similar compounds in the data set.^13^ More to the point, it is important to emphasize that the *in silico* results obtained for the isolated compounds herein are solely derived from *in silico* models, and further experimental studies are necessary to validate these findings.

In the *in silico* phase of our study, isomeranzin was identified as “mutagenic”, yet no research in the literature has addressed the mutagenic activity of isomeranzin. Although isomeranzin was not found to be potent in terms of cytotoxic activity in MCF-7 cells, it was found to be worth studying for cardiovascular analeptic effect in computer-based analyses. Since it was evaluated as mutagenic in *in silico* mutagenicity analyses, it is strongly suggested that confirmatory mutagenicity/genotoxicity tests including Ames test, *in vitro* chromosomal aberration or *in vitro* micronucleus test should be performed in future studies, taking into account the ICH S2 guideline. On the other hand, the compounds 3-methoxy nobiletin, nobiletin and tangeretin were predicted as “nonmutagenic” in *in silico* analysis. In a previous study, a flavonoid mixture isolated from *Citrus* peels (comprising nobiletin (32.5%), tangeretin (14%), and eight other flavonoids) was evaluated for mutagenicity using the Ames test and L5178Y tk ± mouse lymphoma cells. The results demonstrated no mutagenic activity, either in the presence or absence of metabolic activation.^60^ Furthermore, the antimutagenic effects of both tangeretin and nobiletin were experimentally demonstrated,^61^ suggesting that these metabolites could be utilized for chemopreventive purposes in cancer. The findings of the previous studies support the *in silico* data obtained for nobiletin, which exhibited the highest *in vitro* cytotoxic activity in the present study.

## 5 Conclusion

*Citrus* fruits are economically significant plants cultivated in all tropical and subtropical countries. They produce fruits of high importance due to their global accessibility, popularity, and contributions to human health and diet. Literature studies reveal that fruits of *Citrus* species are widely used for the prevention and treatment of various diseases. The higher concentration of bioactive secondary metabolites in fruit peels and the potential to valorize *Citrus* waste into high-value products have prompted the design of our study to focus on *Citrus* fruit peels. Since the MCF-7 cell line was found to respond well to the extract, fraction, subfraction, and nobiletin from *C. aurantium*, further research is planned to assess their effects on the estrogen-positive *in vivo* breast cancer model in order to obtain data related to histopathology, immunohistochemistry, biochemistry, and gene-level mechanistic approaches. Further preclinical and phytochemical studies can also be conducted on the extracts of other *Citrus* species (*C. grandis*, *C. medica,* and *C. paradisi*) that we evaluated for their effects, which also show cytotoxic activity. Moreover, since *Citrus* fruit peel waste contains potential cytotoxic compounds that have diverse molecular structures and high therapeutic value, it is suggested that efforts should be made to convert this waste into a resource that will provide economic benefits. Additionally, research should focus on the development plant-based formulations with standardized peel extract of *C. aurantium* as well as various production processes to obtain bioactive secondary metabolites, particularly nobiletin.

## Supporting Information Available

The Supporting Information consist of spectral data and NMR data table for isolated compounds together with detailed cytotoxic activity figures (PDF)
