# Supplementary material for: Cytotoxic Effects of Citrus Peels on Breast Tumor: Opportunities for Waste to Raw Material Conversion
Source: ACS Omega. 2025 Apr 17;10(16):16900–8. doi: 10.1021/acsomega.5c01170 (PMC12044562; doi:10.1021/acsomega.5c01170)
Supplement: Supplementary file 1 — ao5c01170_si_001.pdf [file ao5c01170_si_001.pdf]

Supporting Information

Cytotoxic effects of *Citrus* peels on breast tumor: Opportunities for waste to raw material conversion

Ömer Faruk YAKINCI[1,2], Esra EMERCE[3], Perihan GÜRBÜZ[4,*],
Mürşide Ayşe DEMİREL[5], Songül ÇERİBAŞI[6], İpek SÜNTAR[7,*]

[1]National Poisons Information Service, Republic of Türkiye Ministry of Health, Ankara, Türkiye
[2]Institute of Health Sciences, Gazi University, Ankara, Türkiye
[3]Department of Pharmaceutical Toxicology, Faculty of Pharmacy, Gazi University, Ankara, Türkiye
[4]Department of Pharmacognosy, Faculty of Pharmacy, Erciyes University, Kayseri, Türkiye
[5]Department of Pharmaceutical Basic Sciences, Faculty of Pharmacy, Gazi University, Ankara, Türkiye
[6]Department of Pathology, Faculty of Veterinary Medicine, Fırat University, Elazığ, Türkiye
[7]Department of Pharmacognosy, Faculty of Pharmacy, Gazi University, Ankara, Türkiye
*Correspondence: pgurbuz@erciyes.edu.tr; ipesin@gazi.edu.tr

List of Supporting Information

HRESIMS, and NMR spectra of compounds CAS-5, CAS-10, CAS-11, CAS-12

Isomeranzin (CAS-5)

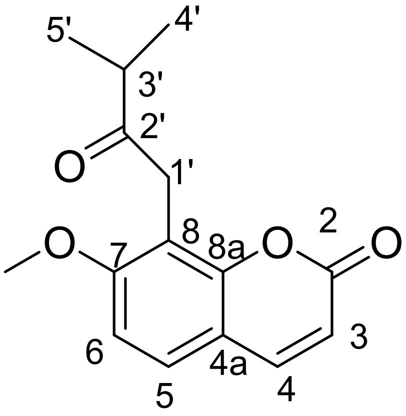

Figure	Caption	page
**Figure S1.**	HRESIMS (pos.) spectrum of **CAS-5**	3
**Figure S2.**	^{1}H NMR spectrum (600 MHz, $CDCl_3$) of **CAS-5**	4
**Figure S3.**	^{13}C NMR spectrum (150 MHz, $CDCl_3$) of **CAS-5**	5
**Figure S4.**	HSQC NMR spectrum ($CDCl_3$, 600 MHz, 150 MHz) of **CAS-5**	6
**Figure S5.**	HMBC NMR spectrum ($CDCl_3$, 600 MHz, 150 MHz) of **CAS-5**	7
**Table S1.**	Assignments of ^{1}H NMR and ^{13}C NMR signals ($CDCl_3$, ^{13}C: 150 MHz; ^{1}H: 600 MHz) for **CAS-5**	8

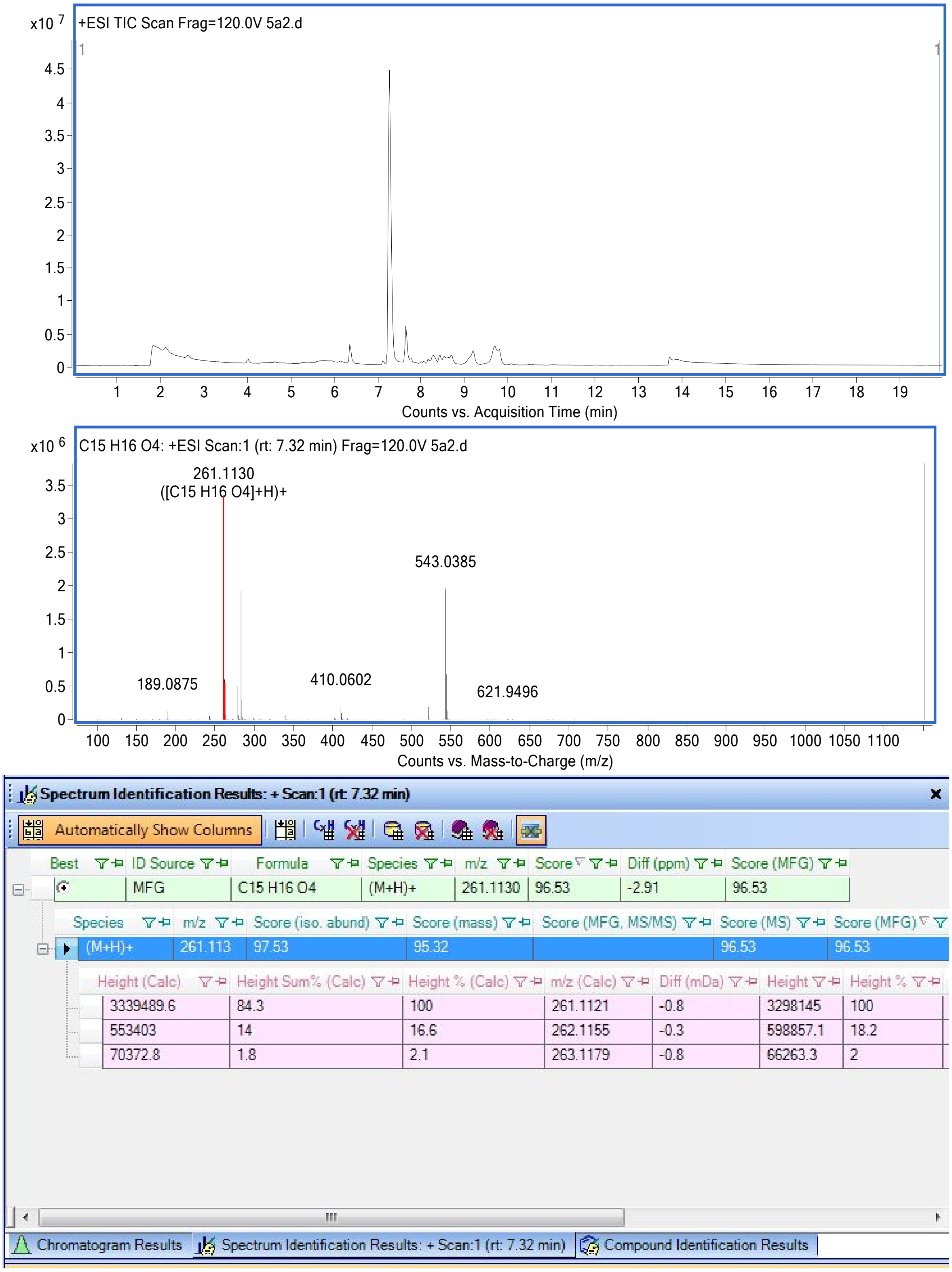

Figure S1. HRESIMS (pos.) spectrum of **CAS-5**

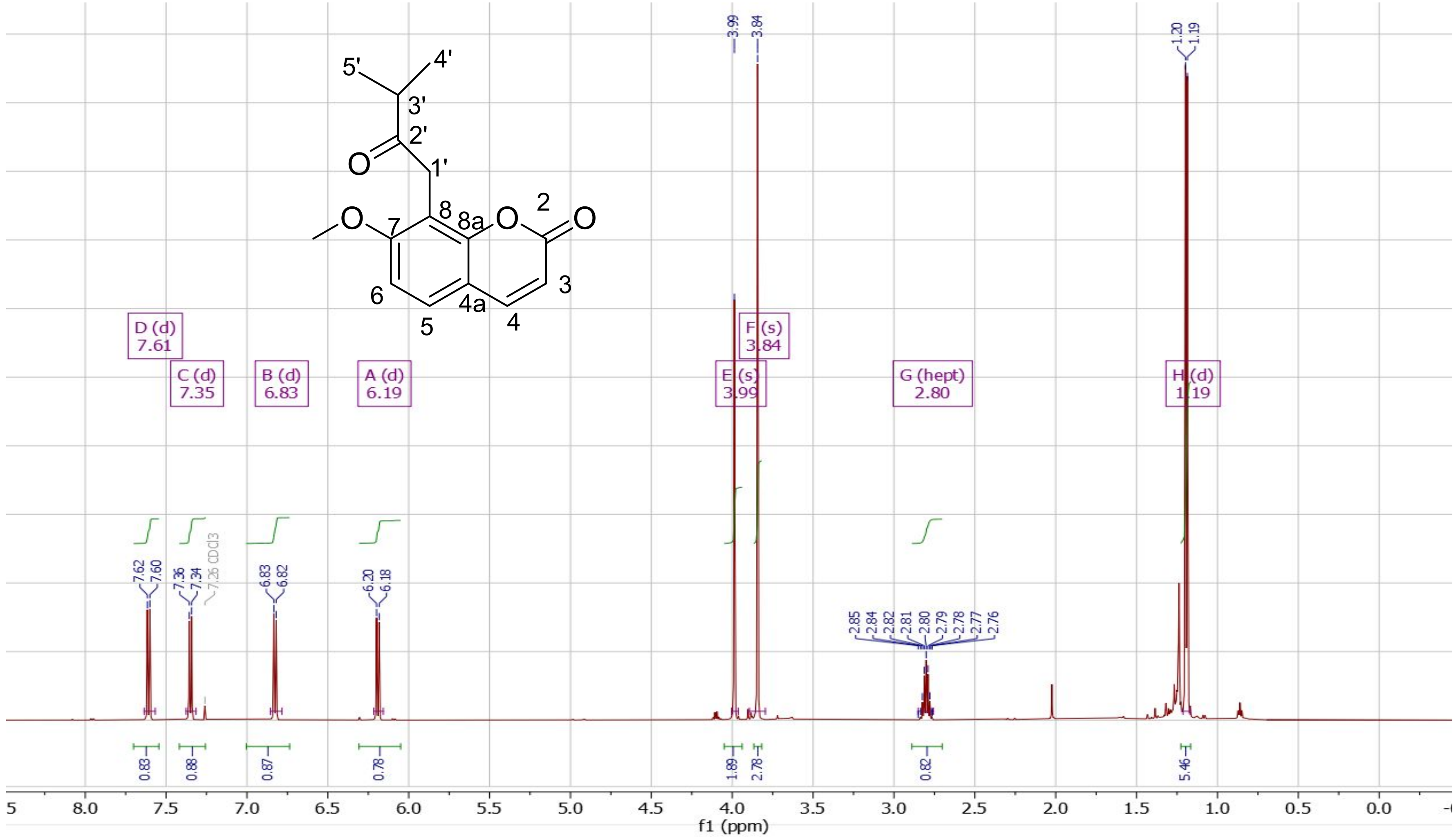

Figure S2. ^{1}H NMR spectrum (600 MHz, $CDCl_3$) of **CAS-5**

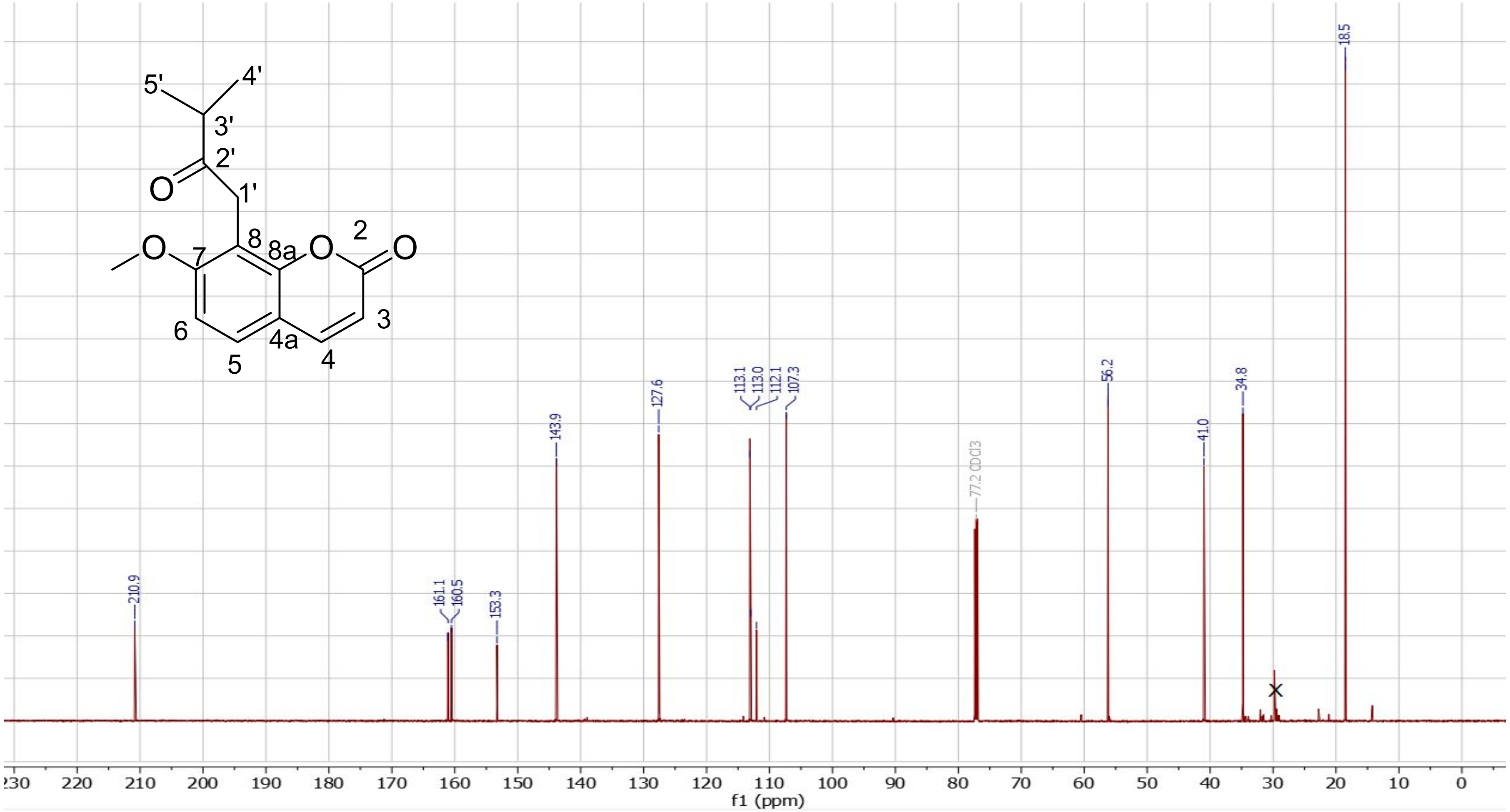

Figure S3. 13C NMR spectrum (150 MHz, $CDCl_3$) of **CAS-5**

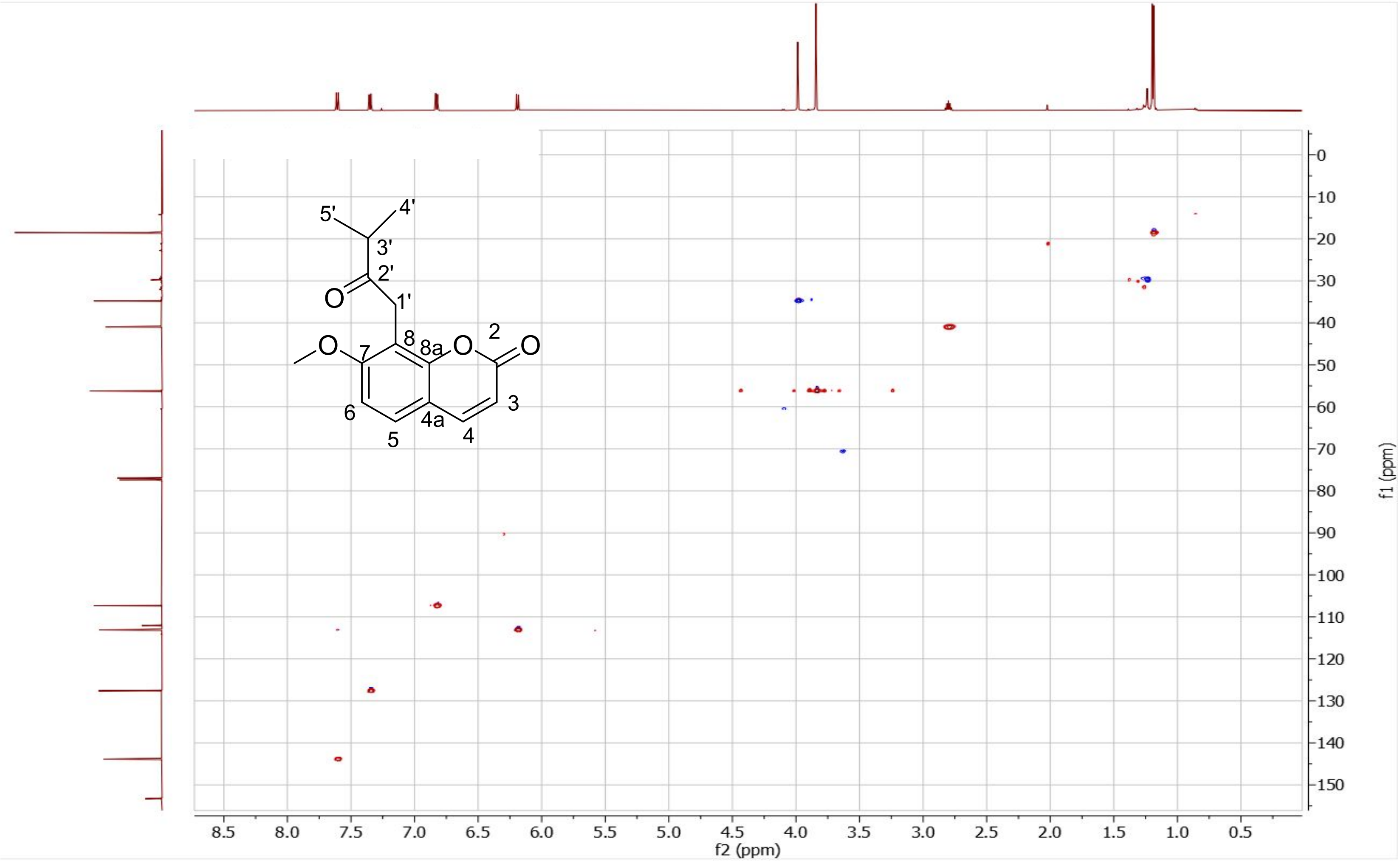

Figure S.4. HSQC NMR spectrum ($CDCl_3$, 600 MHz, 150 MHz) of **CAS-5**

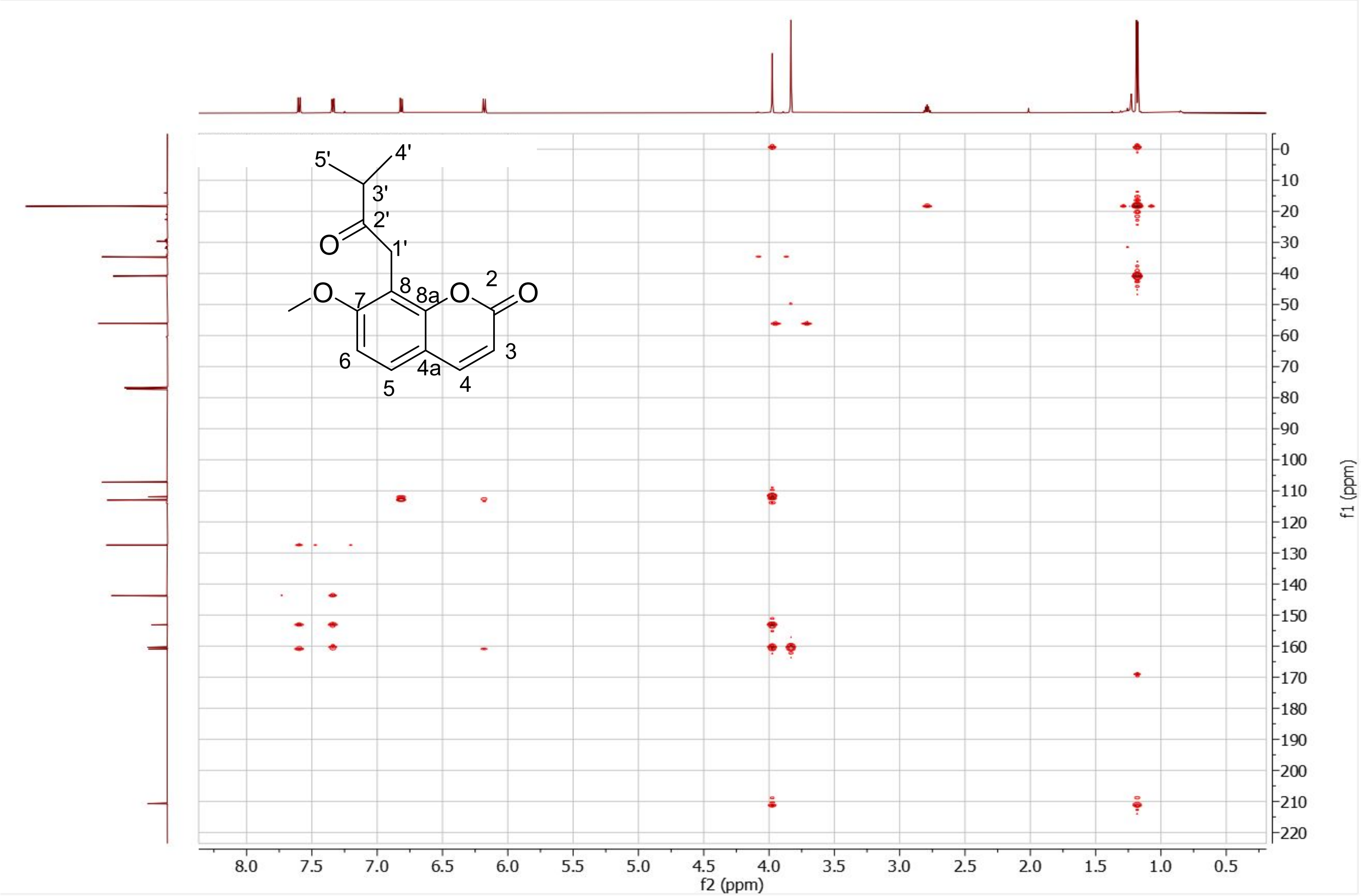

Figure S.5. HMBC NMR spectrum ($CDCl_3$, 600 MHz, 150 MHz) of **CAS-5**

Table S.1. Assignments of ^{1}H NMR and ^{13}C NMR signals ($CDCl_3$, ^{13}C: 150 MHz; ^{1}H: 600 MHz) for **CAS-5**

**C No**	**C/H**	**C (δ in ppm)**	**H (δ in ppm, *J* in Hz)**
**2**	C	161.1	
**3**	CH	143.9	6.19 (d, *J* = 9.5)
**4**	CH	113.1	7.61 (d, *J* = 9.4)
**4a**	C	112.1	
**5**	CH	127.6	7.35 (d, *J* = 8.6)
**6**	CH	107.3	6.83 (d, *J* = 8.6)
**7**	C	160.5	
**8**	C	113.0	
**8a**	C	153.3	
**1'**	CH_2	34.8	3.99 (s)
**2'**	C	210.9	
**3'**	CH	41.0	2.80 (hept, *J* = 6.9)
**4'**	CH_3	18.5	
**5'**	CH_3	18.5	1.19 (d, *J* = 7.0)
**OMe**		56.2	3.84 (s)

3-Metoksi nobiletin (CAS 10)

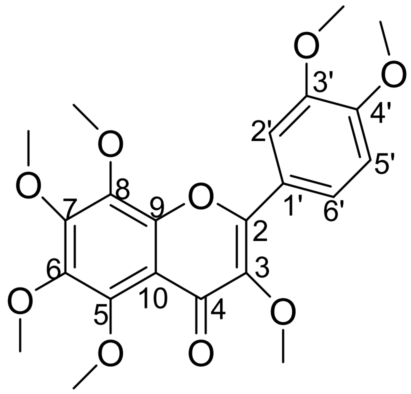

Figure	Caption	page
**Figure S6.**	HRESIMS (pos.) spectrum of **CAS-10**	10
**Figure S7.**	^{1}H NMR spectrum (600 MHz, $CDCl_3$) of **CAS-10**	11
**Figure S8.**	^{13}C NMR spectrum (150 MHz, $CDCl_3$) of **CAS-10**	12
**Figure S9.**	HSQC NMR spectrum ($CDCl_3$, 600 MHz, 150 MHz) of **CAS-10**	13
**Figure S10.**	HMBC NMR spectrum ($CDCl_3$, 600 MHz, 150 MHz) of **CAS-10**	14
**Table S2.**	Assignments of ^{1}H NMR and ^{13}C NMR signals ($CDCl_3$, ^{13}C: 150 MHz; ^{1}H: 600 MHz) for **CAS-10**	15

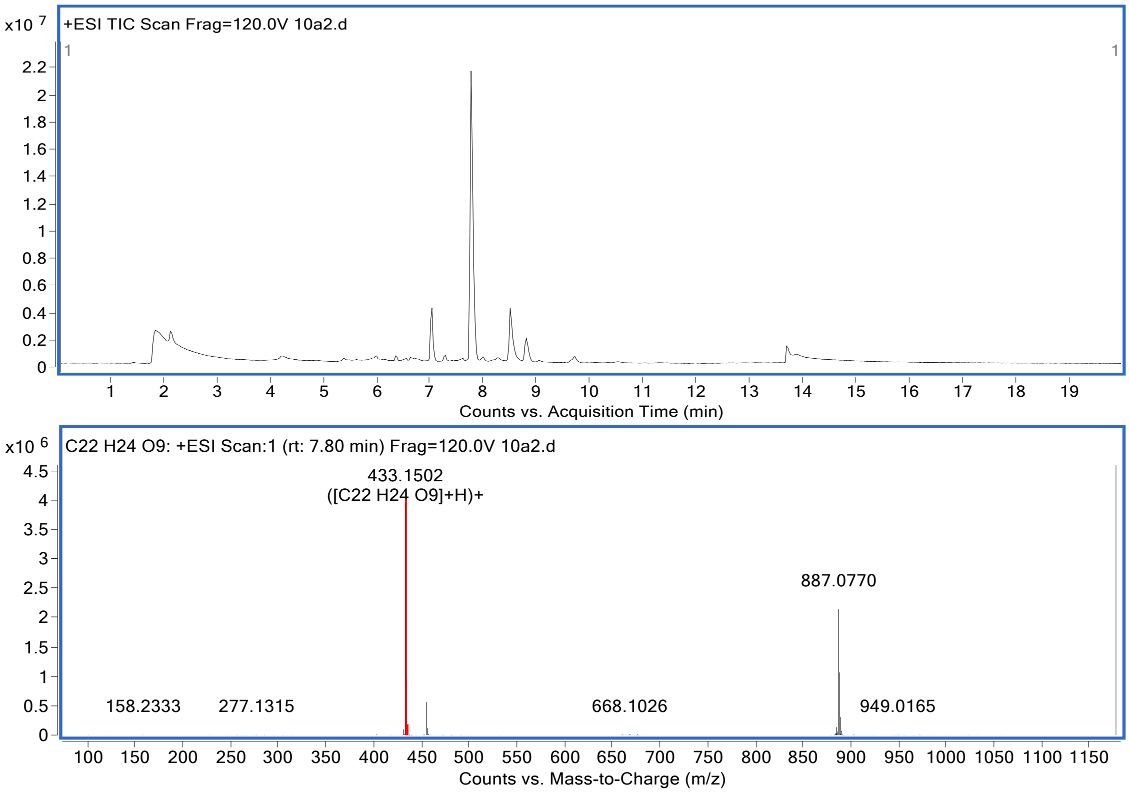

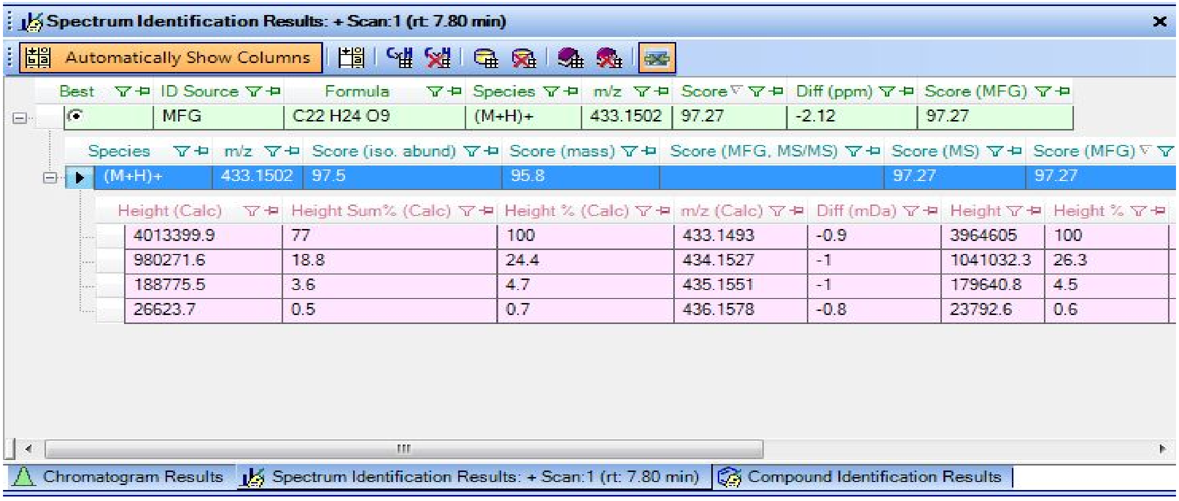

Spectrum Identification Results: + Scan:1 (rt: 7.80 min)

Automatically Show Columns

Best	ID Source	Formula	Species	m/z	Score	Diff (ppm)	Score (MFG)
◉	MFG	C22 H24 O9	(M+H)+	433.1502	97.27	-2.12	97.27

Species	m/z	Score (iso. abund)	Score (mass)	Score (MFG, MS/MS)	Score (MS)	Score (MFG)
(M+H)+	433.1502	97.5	95.8		97.27	97.27

Height (Calc)	Height Sum% (Calc)	Height % (Calc)	m/z (Calc)	Diff (mDa)	Height	Height %
4013399.9	77	100	433.1493	-0.9	3964605	100
980271.6	18.8	24.4	434.1527	-1	1041032.3	26.3
188775.5	3.6	4.7	435.1551	-1	179640.8	4.5
26623.7	0.5	0.7	436.1578	-0.8	23792.6	0.6

Chromatogram Results | Spectrum Identification Results: + Scan:1 (rt: 7.80 min) | Compound Identification Results

Figure S6. HRESIMS (pos.) spectrum of **CAS-10**

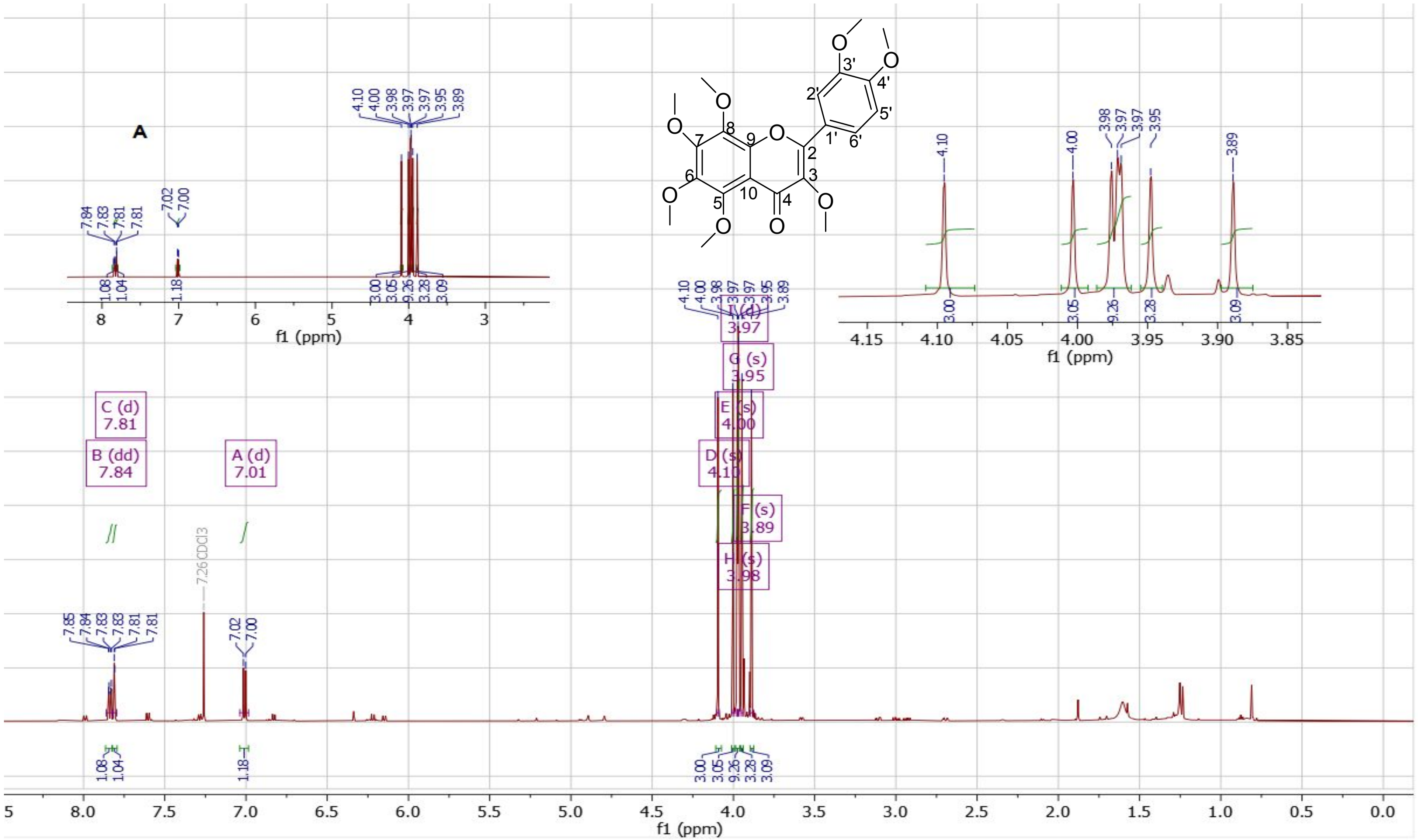

Figure S7. ^{1}H NMR spectrum (600 MHz, $CDCl_3$) of **CAS-10**

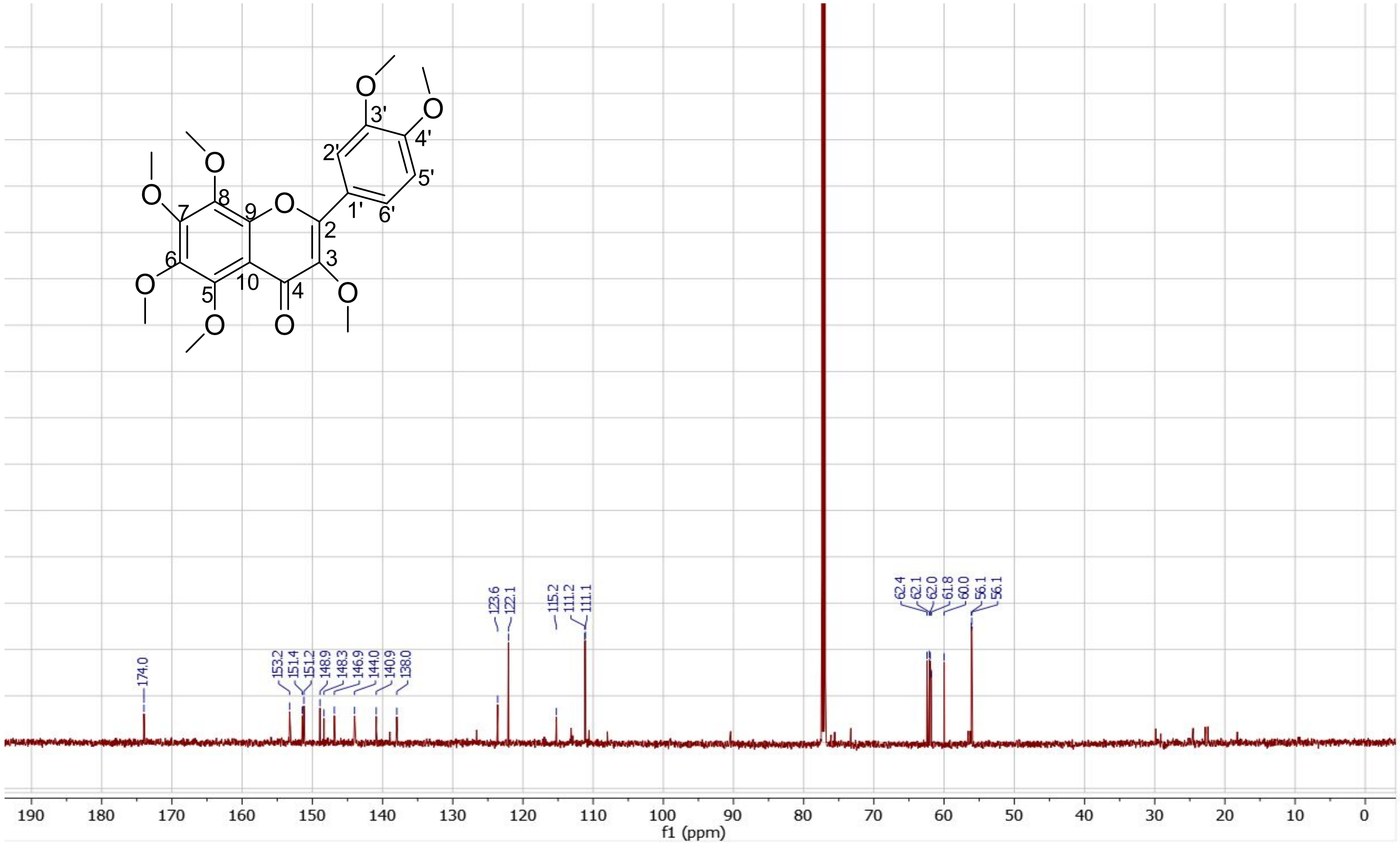

Figure S8. ^{13}C NMR spectrum (150 MHz, $CDCl_3$) of **CAS-10**

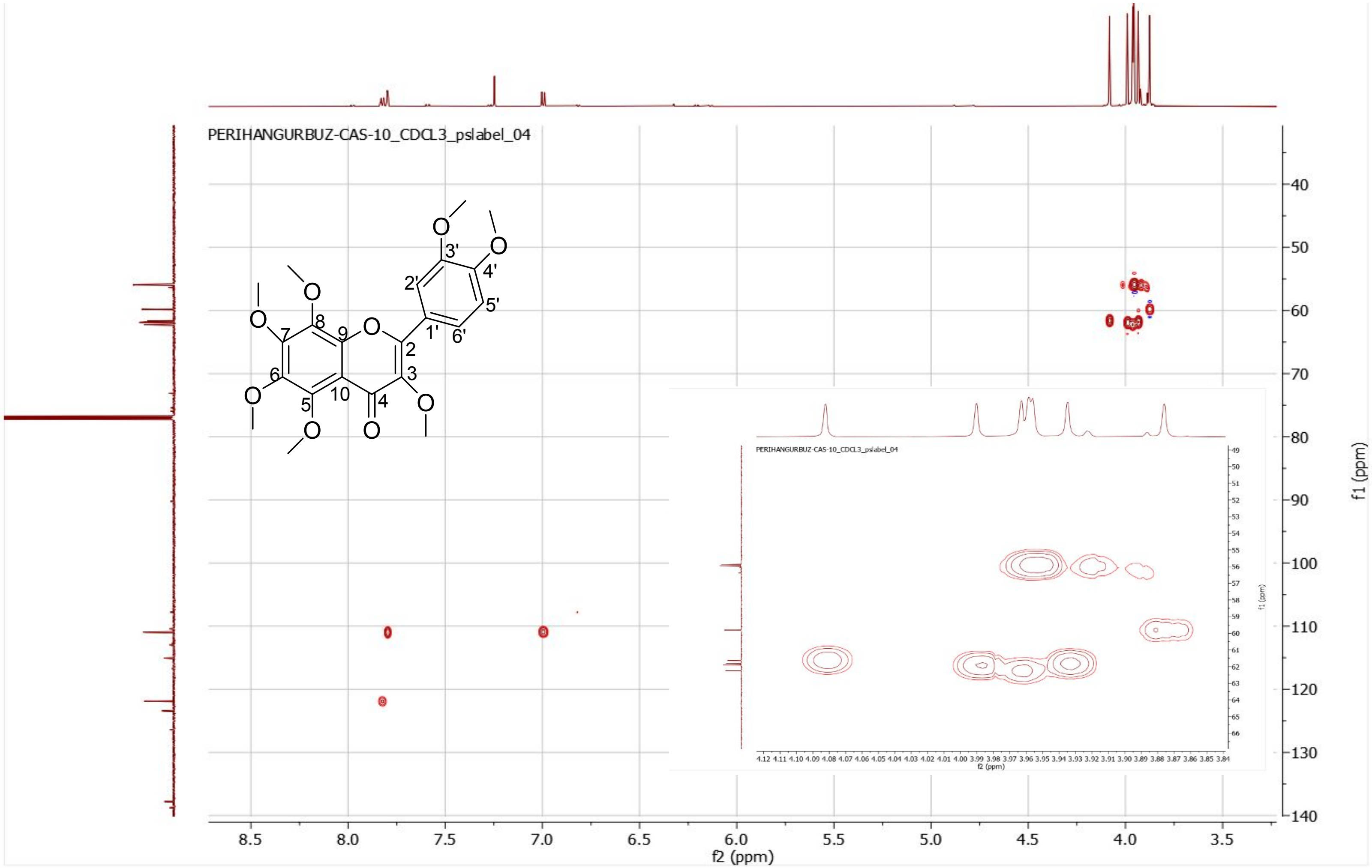

Figure S9. HSQC NMR spectrum ($CDCl_3$, 600 MHz, 150 MHz) of **CAS-10**

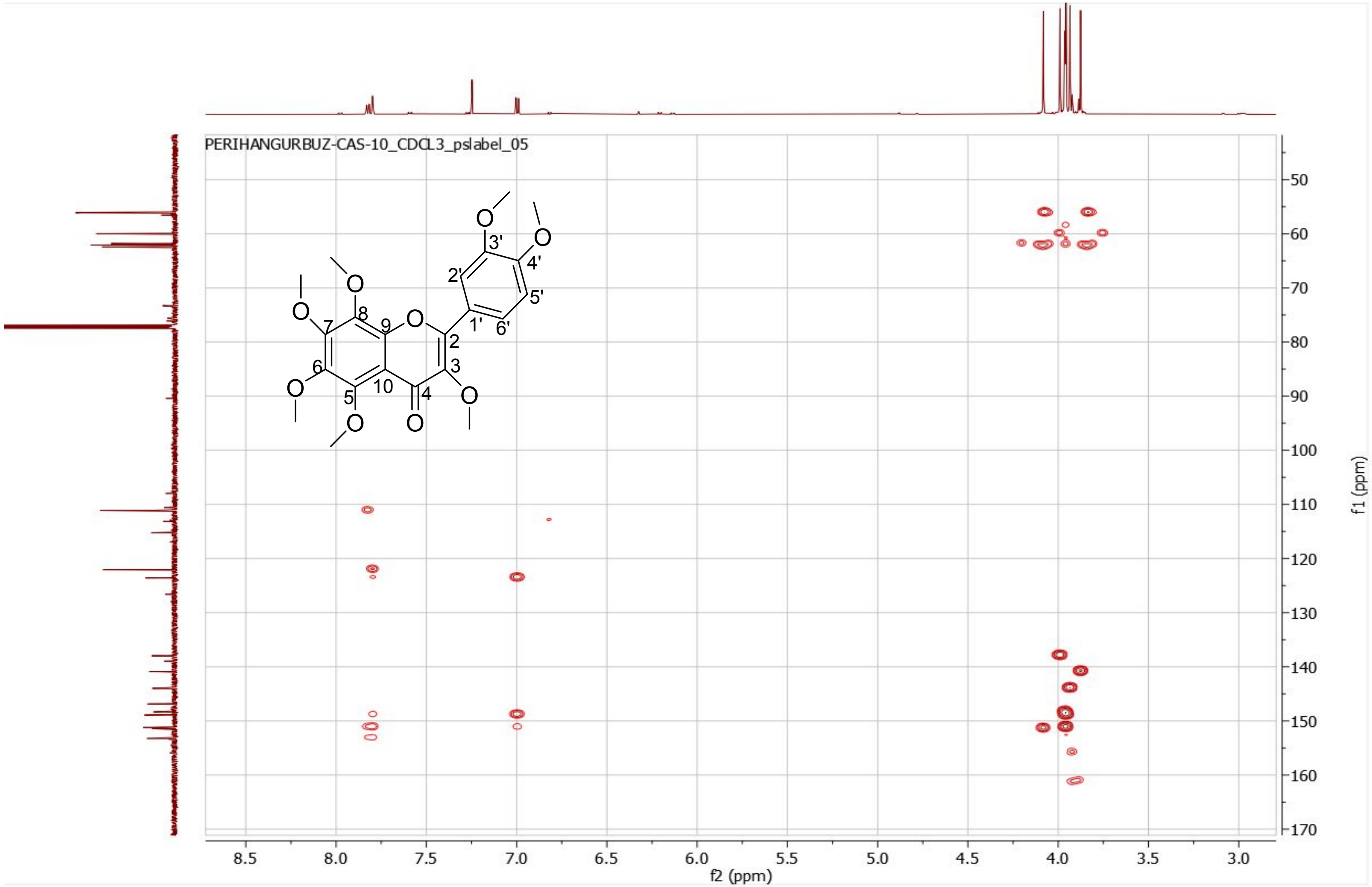

Figure S10. HMBC NMR spectrum ($CDCl_3$, 600 MHz, 150 MHz) of **CAS-10**

Table S2. Assignments of ^{1}H NMR and ^{13}C NMR signals ($CDCl_3$, ^{13}C: 150 MHz; ^{1}H: 600 MHz) for **CAS-10**

**C No**	**C/H**	**C (δ in ppm)**	**H (δ in ppm, *J* in Hz)**
**2**	C	153.2	
**3**	C	140.9	
**4**	C	174.0	
**5**	C	144.0	
**6**	C	138.0	
**7**	C	151.4	
**8**	C	148.3	
**9**	C	146.9	
**10**	C	115.2	
**1'**	C	123.6	
**2'**	CH	111.1	7.81 (d, *J* = 2.1)
**3'**	C	148.9	
**4'**	C	151.2	
**5'**	CH	111.2	7.01 (d, *J* = 8.5)
**6'**	CH	122.1	7.84 (dd, *J* = 8.5, 2.0)
**3-OMe**		60.0	3.89 (s)
**5-OMe**		62.4	3.98 (s)
**6-OMe**		62.1	4.00 (s)
**7-OMe**		62.0	4.10 (s)
**8-OMe**		61.8	3.95 (s)
**3'-OMe**		56.1	3.97 (s)
**4'-OMe**		56.1	3.97 (s)

Nobiletin (CAS 11)

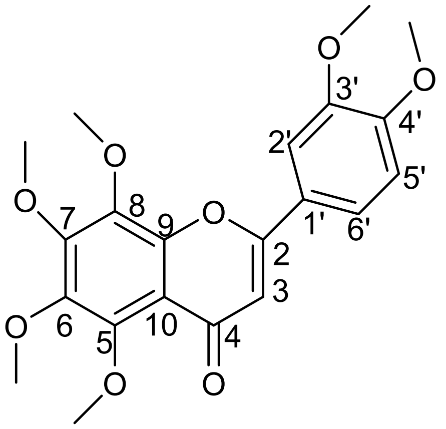

Figure	Caption	page
**Figure S11.**	HRESIMS (pos.) spectrum of **CAS-11**	17
**Figure S12.**	^{1}H NMR spectrum (600 MHz, $CDCl_3$) of **CAS-11**	18
**Figure S13.**	^{13}C NMR spectrum (150 MHz, $CDCl_3$) of **CAS-11**	19
**Figure S14.**	HSQC NMR spectrum ($CDCl_3$, 600 MHz, 150 MHz) of **CAS-11**	20
**Figure S15.**	HMBC NMR spectrum ($CDCl_3$, 600 MHz, 150 MHz) of **CAS-11**	21
**Table S3.**	Assignments of ^{1}H NMR and ^{13}C NMR signals ($CDCl_3$, ^{13}C: 150 MHz; ^{1}H: 600 MHz) for **CAS-11**	22

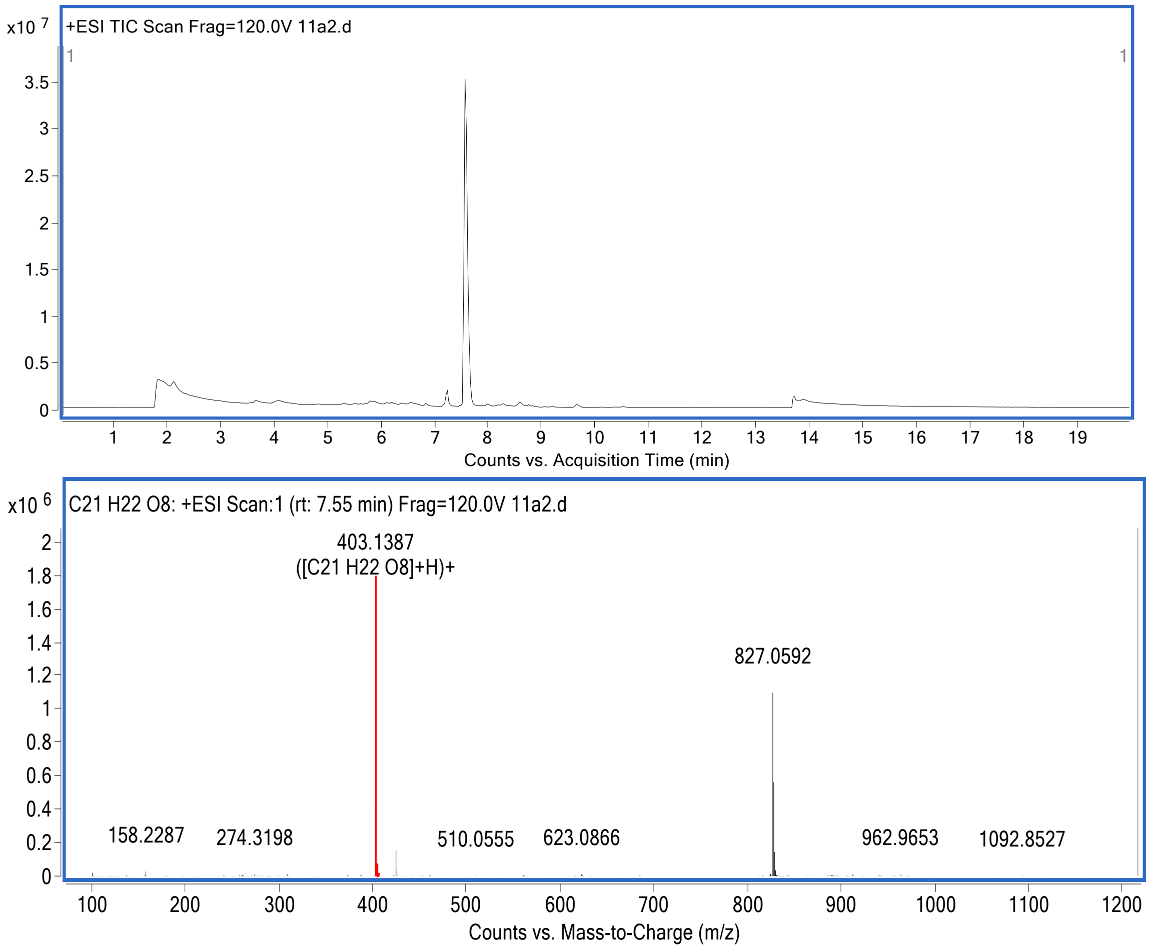

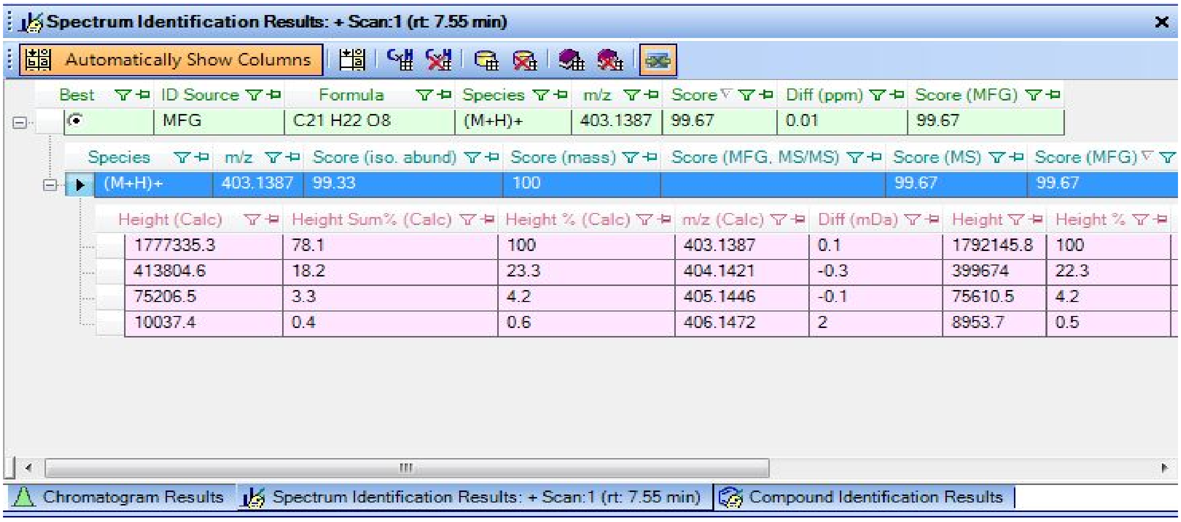
Spectrum Identification Results: + Scan:1 (rt: 7.55 min)

Automatically Show Columns

Best	ID Source	Formula	Species	m/z	Score	Diff (ppm)	Score (MFG)
	MFG	C21 H22 O8	(M+H)+	403.1387	99.67	0.01	99.67

Species	m/z	Score (iso. abund)	Score (mass)	Score (MFG, MS/MS)	Score (MS)	Score (MFG)
(M+H)+	403.1387	99.33	100		99.67	99.67

Height (Calc)	Height Sum% (Calc)	Height % (Calc)	m/z (Calc)	Diff (mDa)	Height	Height %
1777335.3	78.1	100	403.1387	0.1	1792145.8	100
413804.6	18.2	23.3	404.1421	-0.3	399674	22.3
75206.5	3.3	4.2	405.1446	-0.1	75610.5	4.2
10037.4	0.4	0.6	406.1472	2	8953.7	0.5

Chromatogram Results | Spectrum Identification Results: + Scan:1 (rt: 7.55 min) | Compound Identification Results

Figure S11. HRESIMS (pos.) spectrum of **CAS-11**

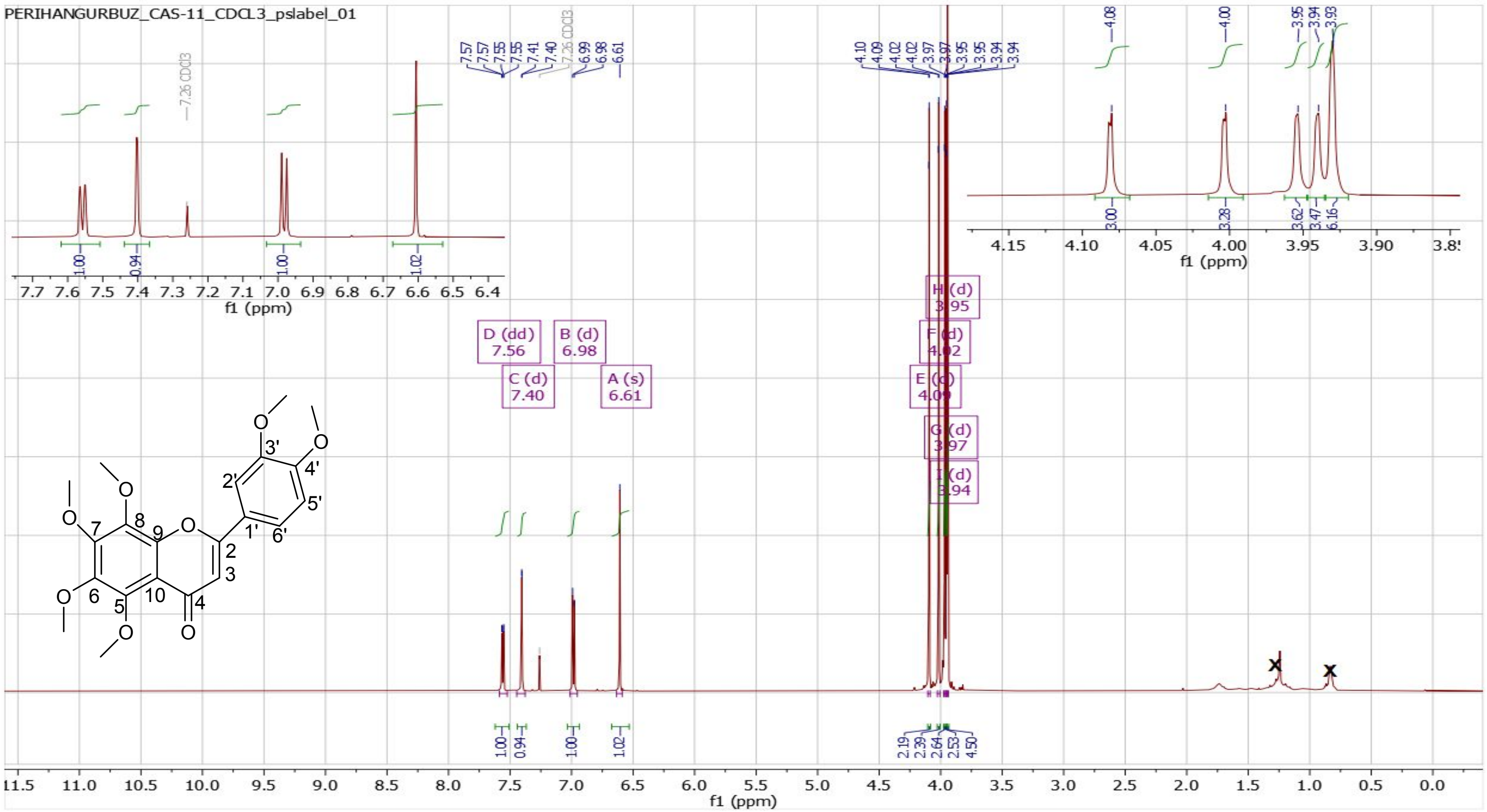

Figure S12. ^{1}H NMR spectrum (600 MHz, $CDCl_3$) of **CAS-11**

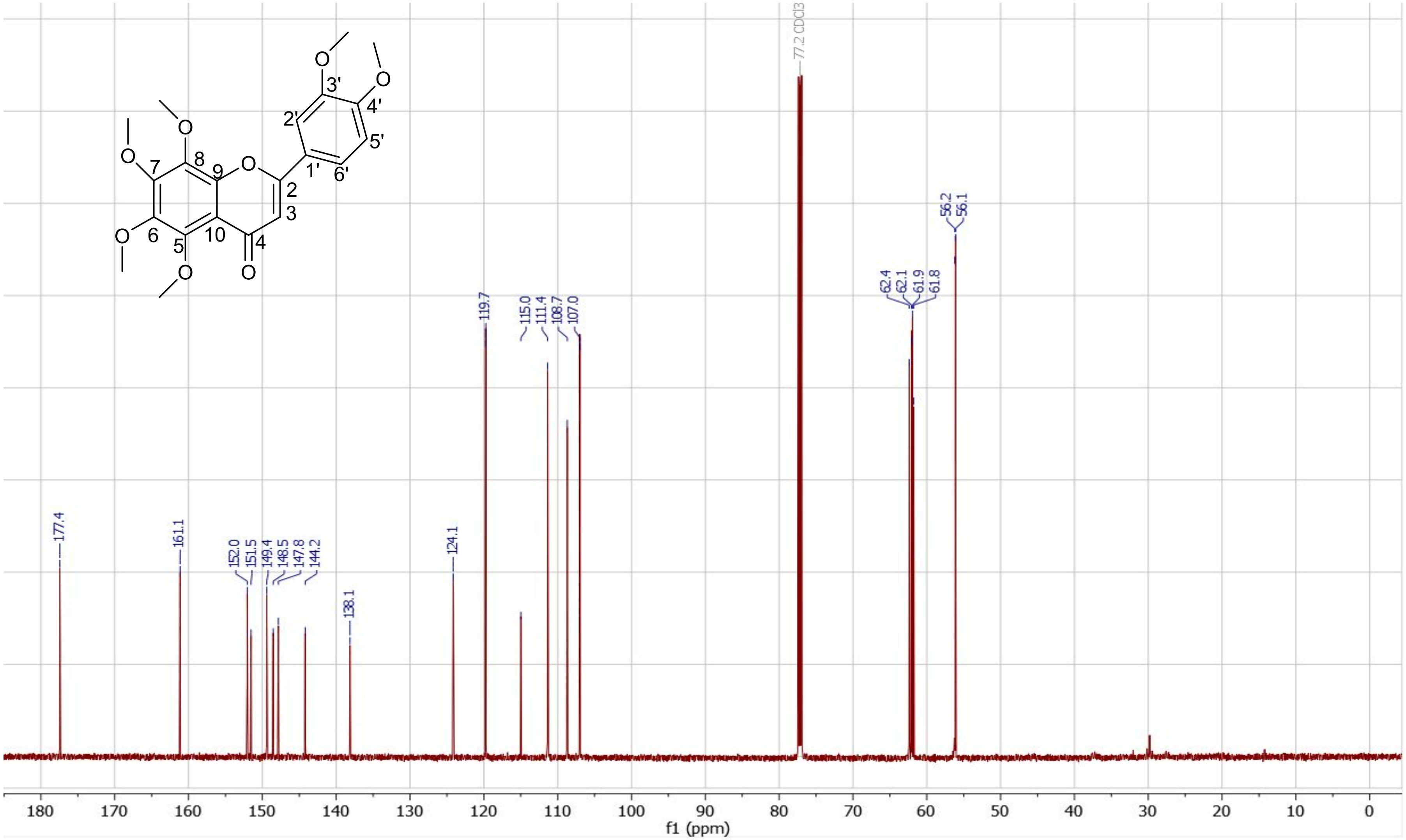

Figure S13. ^{13}C NMR spectrum (150 MHz, $CDCl_3$) of **CAS-11**

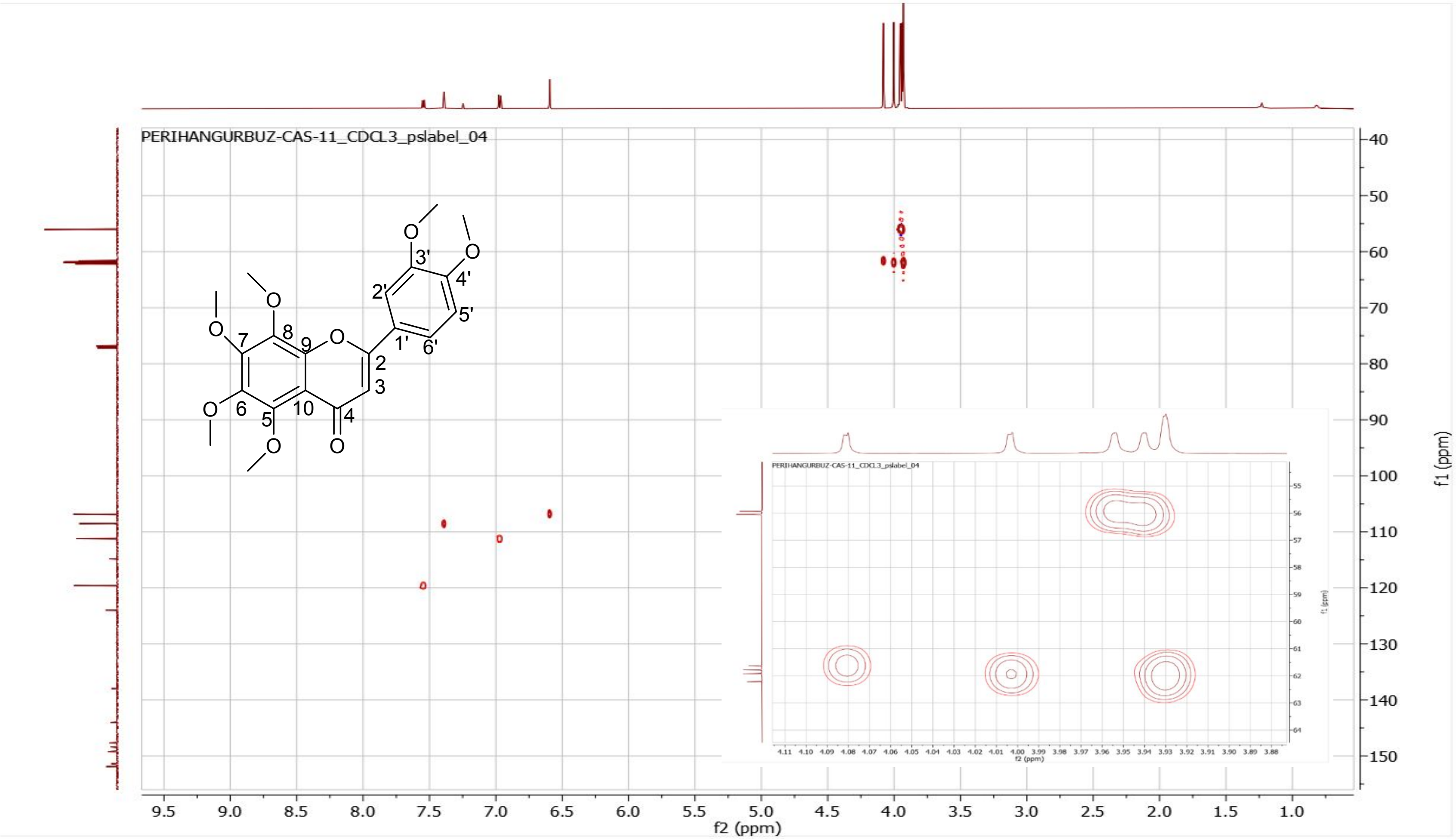

Figure S14. HSQC NMR spectrum ($CDCl_3$, 600 MHz, 150 MHz) of **CAS-11**

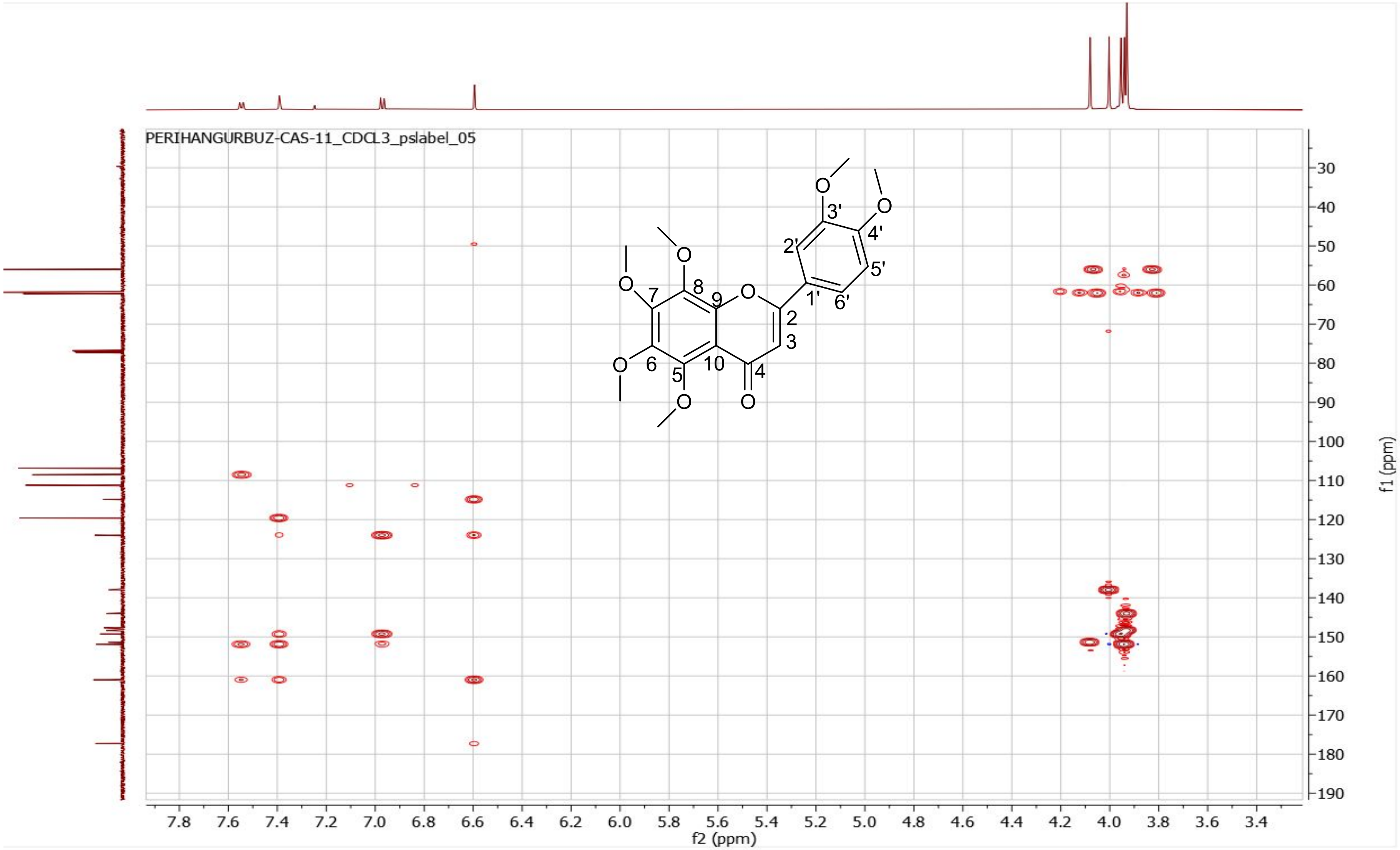

Figure S15. HMBC NMR spectrum ($CDCl_3$, 600 MHz, 150 MHz) of **CAS-11**

Table S3. Assignments of ^{1}H NMR and ^{13}C NMR signals ($CDCl_3$, ^{13}C: 150 MHz; ^{1}H: 600 MHz) for **CAS-11**

C No	C/H	C (δ in ppm)	H (δ in ppm, *J* in Hz)
**2**	C	161.1	
**3**	CH	107.0	6.61 (br s)
**4**	C	177.4	
**5**	C	144.2	
**6**	C	138.1	
**7**	C	151.5	
**8**	C	148.5	
**9**	C	147.8	
**10**	C	115.0	
**1'**	C	124.1	
**2'**	CH	108.7	7.40 (d, *J* = 2.2)
**3'**	C	149.4	
**4'**	C	152.0	
**5'**	CH	111.4	6.98 (d, *J* = 8.5)
**6'**	CH	119.7	7.56 (dd, *J* = 8.4, 2.2)
**5-OMe**		62.4	4.00 (s)
**6-OMe**		62.1	3.93 (s)
**7-OMe**		61.9	3.93 (s)
**8-OMe**		61.8	4.08 (s)
**3'-OMe**		56.2	3.94 (s)
**4'-OMe**		56.1	3.95 (s)

Tangeretin (CAS 12)

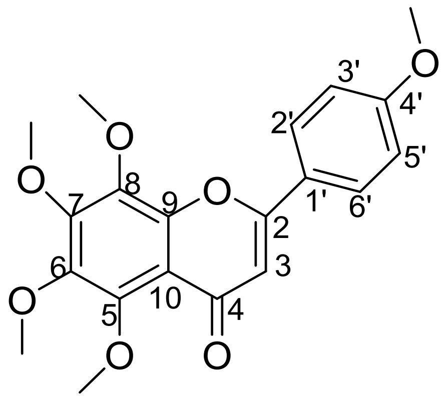

Figure	Caption	page
**Figure S16.**	HRESIMS (pos.) spectrum of **CAS-12**	24
**Figure S17.**	^{1}H NMR spectrum (600 MHz, $CDCl_3$) of **CAS-12**	25
**Figure S18.**	^{13}C NMR spectrum (150 MHz, $CDCl_3$) of **CAS-12**	26
**Figure S19.**	HSQC NMR spectrum ($CDCl_3$, 600 MHz, 150 MHz) of **CAS-12**	27
**Figure S20.**	HMBC NMR spectrum ($CDCl_3$, 600 MHz, 150 MHz) of **CAS-12**	28
**Table S4.**	Assignments of ^{1}H NMR and ^{13}C NMR signals ($CDCl_3$, ^{13}C: 150 MHz; ^{1}H: 600 MHz) for **CAS-12**	29

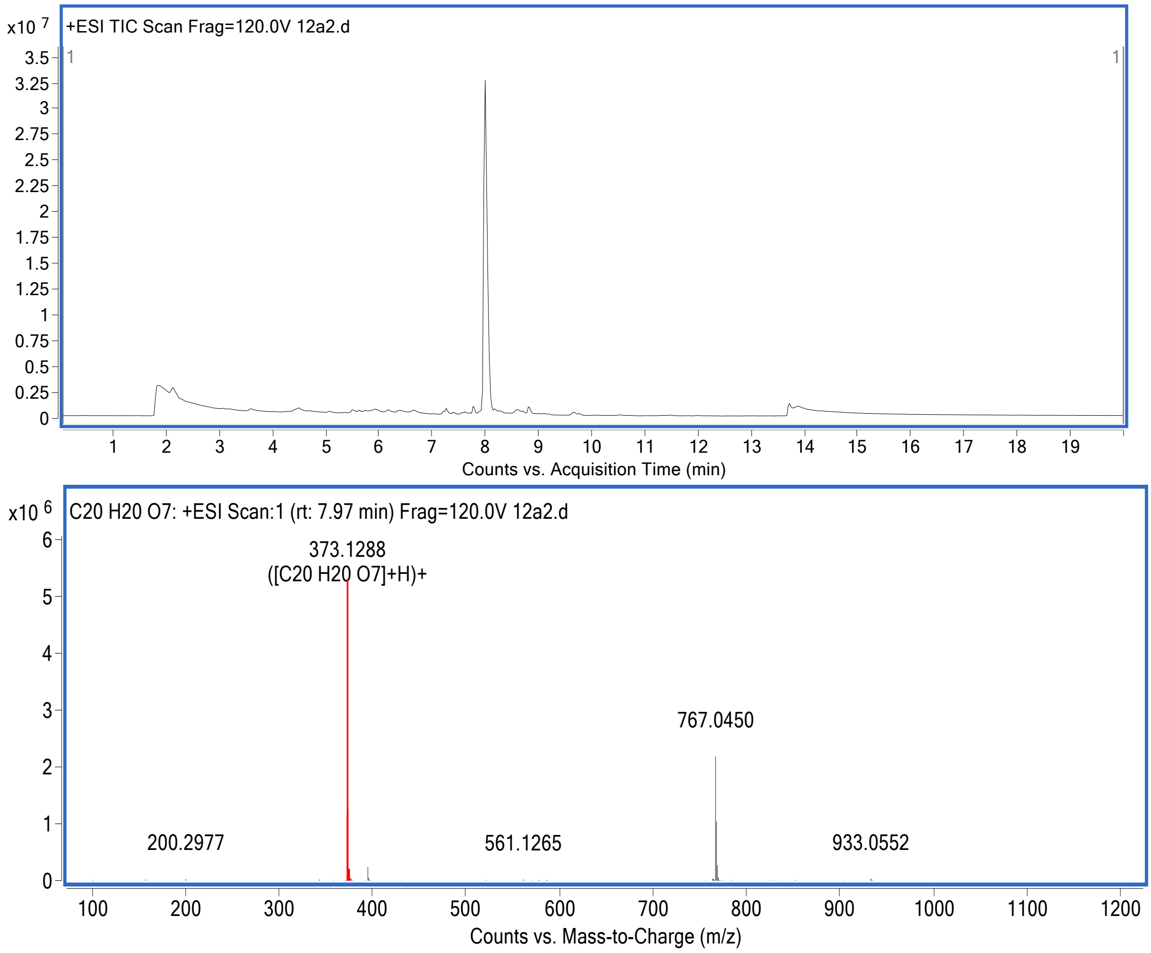

Spectrum Identification Results: + Scan:1 (rt: 7.97 min)

Automatically Show Columns

Best	ID Source	Formula	Species	m/z	Score	Diff (ppm)	Score (MFG)
⊙	MFG	C20 H20 O7	(M+H)+	373.1288	98.23	-1.32	98.23

Species	m/z	Score (iso. abund)	Score (mass)	Score (MFG, MS/MS)	Score (MS)	Score (MFG)
(M+H)+	373.1288	96.91	98.56		98.23	98.23

Height (Calc)	Height Sum% (Calc)	Height % (Calc)	m/z (Calc)	Diff (mDa)	Height	Height %
5311514	79.1	100	373.1282	-0.6	5218870.5	100
1175949.6	17.5	22.1	374.1316	-0.2	1264634.6	24.2
200364.5	3	3.8	375.134	-0.2	202756.1	3.9
25162.1	0.4	0.5	376.1367	0.2	26728.9	0.5

Chromatogram Results | Spectrum Identification Results: + Scan:1 (rt: 7.97 min) | Compound Identification Results

Figure S16. HRESIMS (pos.) spectrum of **CAS-12**

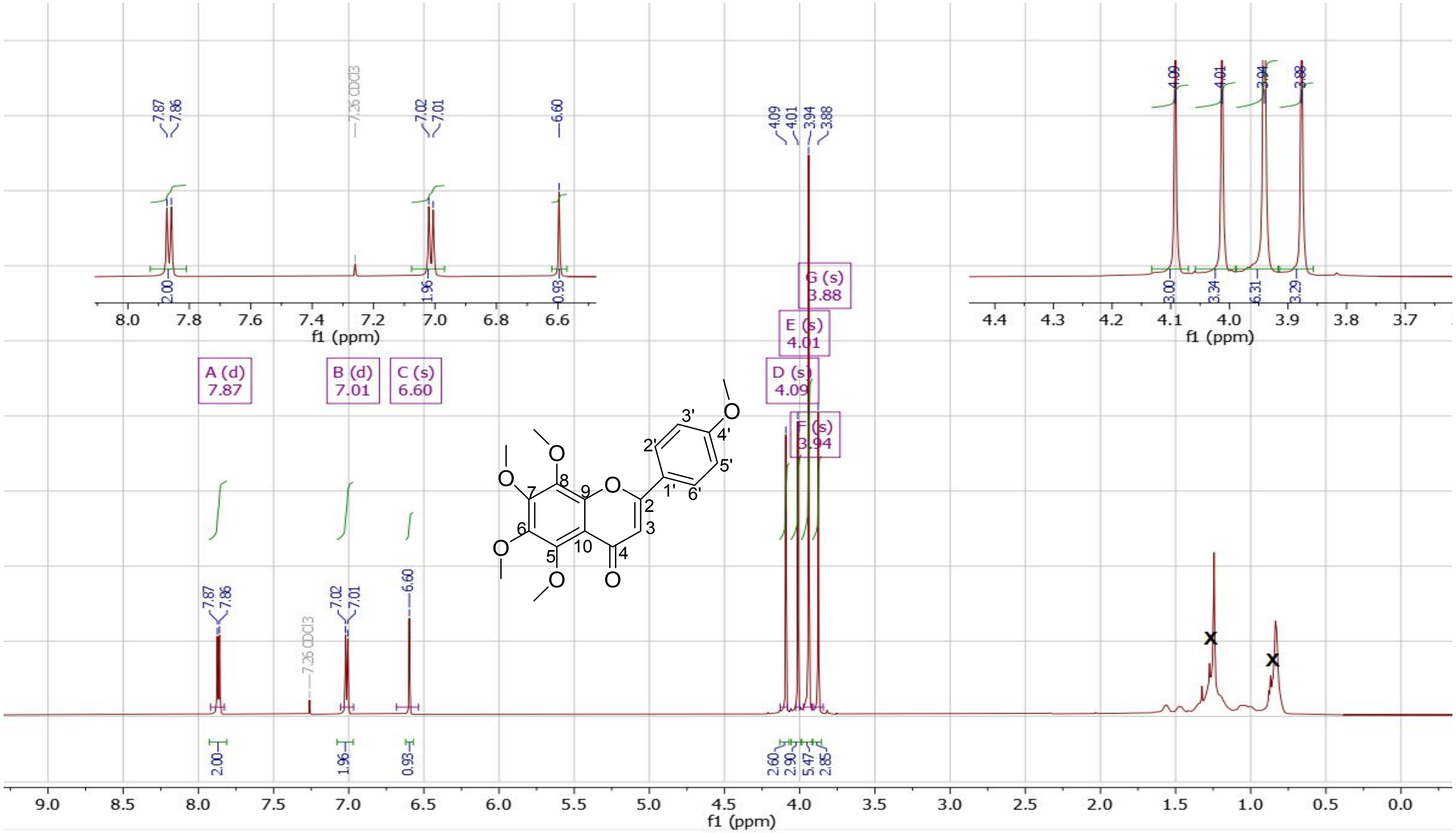

Figure S17. ^{1}H NMR spectrum (600 MHz, $CDCl_3$) of **CAS-12**

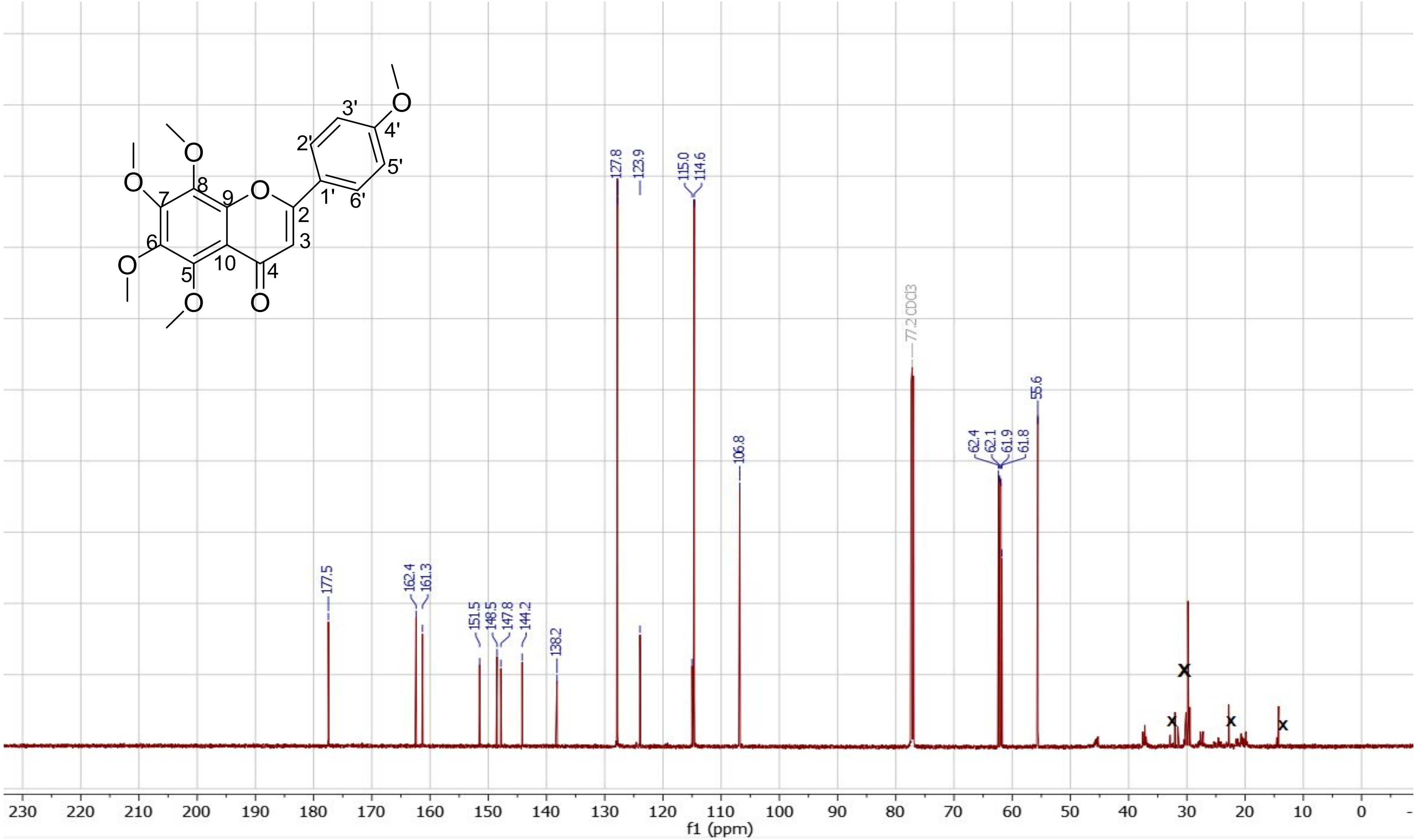

Figure S18. ^{13}C NMR spectrum (150 MHz, $CDCl_3$) of **CAS-12**

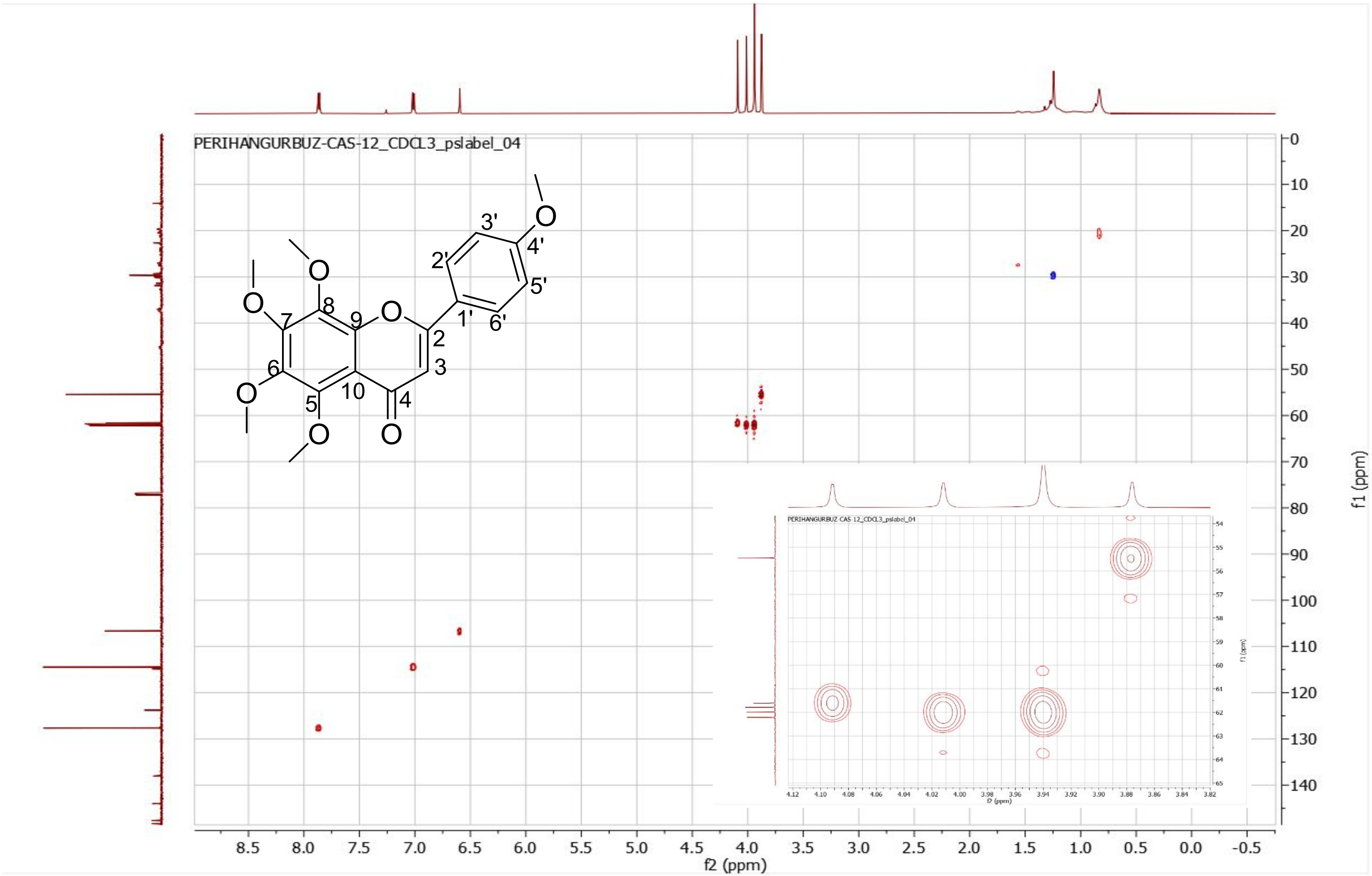

Figure S19. HSQC NMR spectrum ($CDCl_3$, 600 MHz, 150 MHz) of **CAS-12**

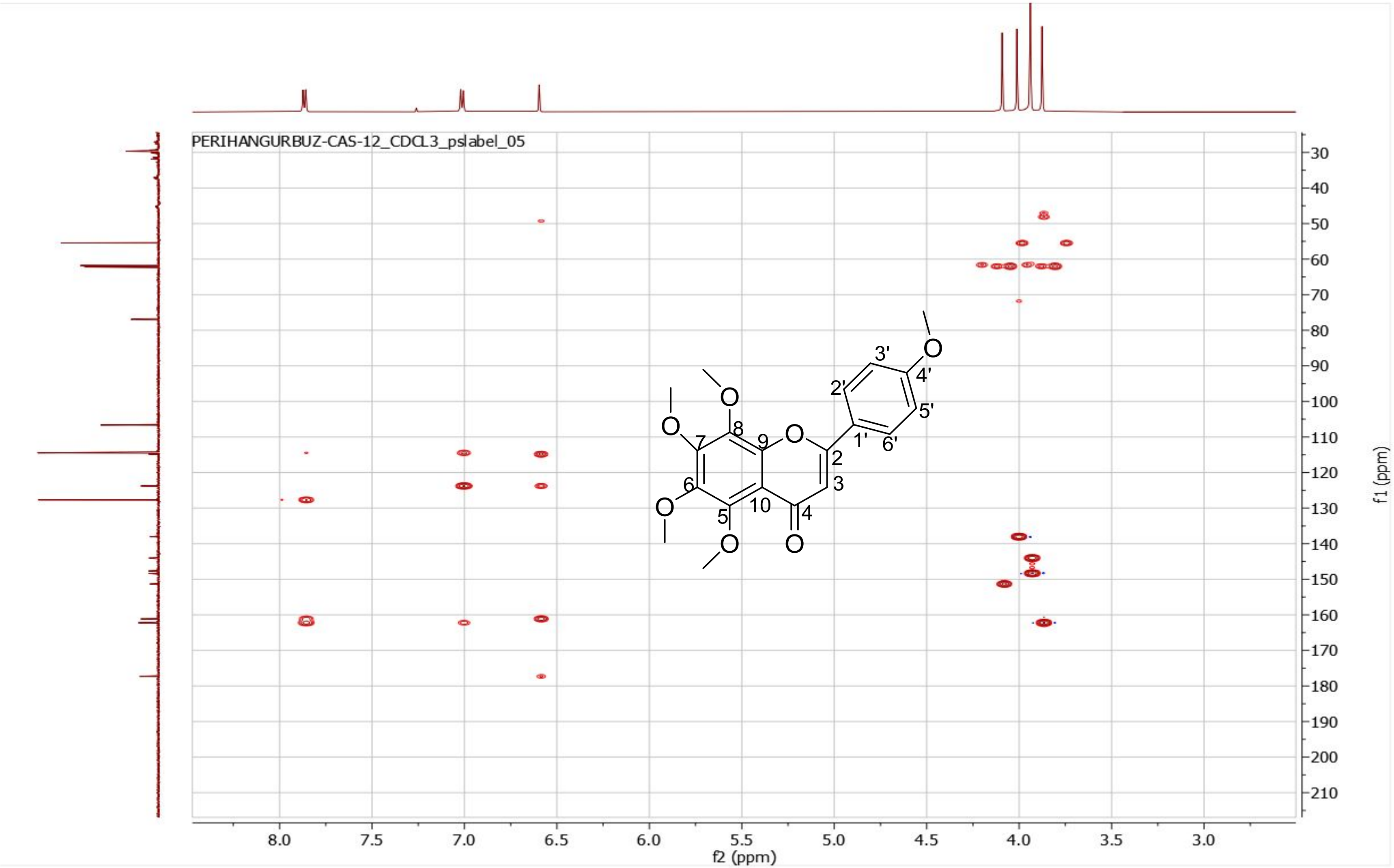

Figure S20. HMBC NMR spectrum ($CDCl_3$, 600 MHz, 150 MHz) of **CAS-12**

Table S4. Assignments of ^{1}H NMR and ^{13}C NMR signals ($CDCl_3$, ^{13}C: 150 MHz; ^{1}H: 600 MHz) for **CAS-12**

C No	C/H	C (δ in ppm)	H (δ in ppm, *J* in Hz)
**2**	C	161.3	
**3**	CH	106.8	6.60 (s)
**4**	C	177.5	
**5**	C	144.2	
**6**	C	138.2	
**7**	C	151.5	
**8**	C	148.5	
**9**	C	147.8	
**10**	C	115.0	
**1'**	C	123.9	
**2'**	CH	127.8	7.87 (d, *J* = 8.6)
**3'**	CH	114.6	7.01 (d, *J* = 8.7)
**4'**	C	162.4	
**5'**	CH	114.6	7.01 (d, *J* = 8.7)
**6'**	CH	127.8	7.87 (d, *J* = 8.6)
**5-OMe**		62.4	3.94 (s)
**6-OMe**		61.8	4.09 (s)
**7-OMe**		62.1	4.01 (s)
**8-OMe**		61.9	3.94 (s)
**4'-OMe**		55.6	3.88 (s)

Cytotoxic Activity		
**Figure**	**Caption**	**page**
**Figure S21.**	Cytotoxic effects of Citrus EtOAc extracts on the MCF-7 cell line	30
**Figure S22.**	Cytotoxic effects of the SP LH-20 column fractions of the CA EtOAc extract on the MCF-7 (A) and MCF-10A (B) cell lines	31
**Figure S23.**	Cytotoxic effects of the silica gel column fractions (sub-fractions) on the MCF-7 (A) and MCF-10A (B) cell lines	32
**Figure S24.**	Cytotoxic effects of compounds isolated from CASK 3 on the MCF-7 (A) and MCF-10A (B) cell lines	33

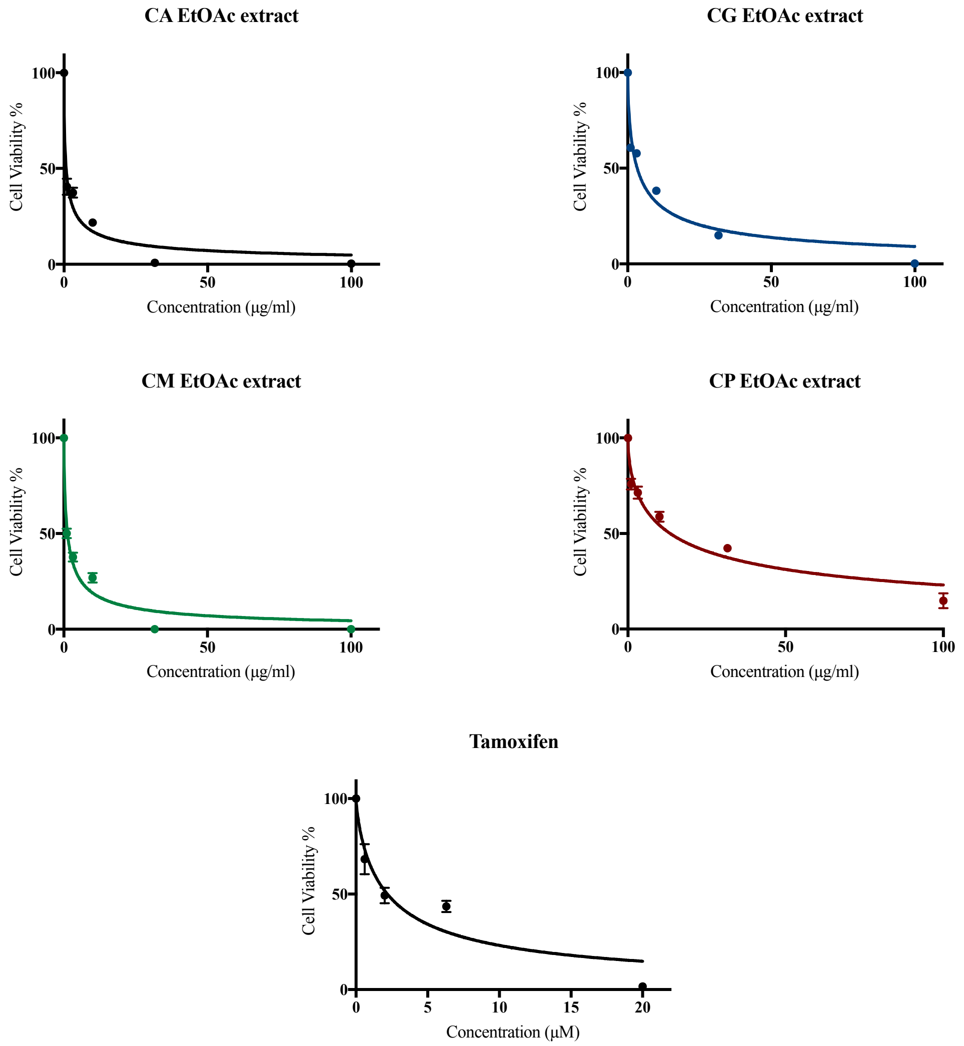

Figure S21. Cytotoxic effects of *Citrus* EtOAc extracts on the MCF-7 cell line. Cells were treated with ethy acetate extracts of *Citrus* species in 48 h and % cell viabilities were measured.

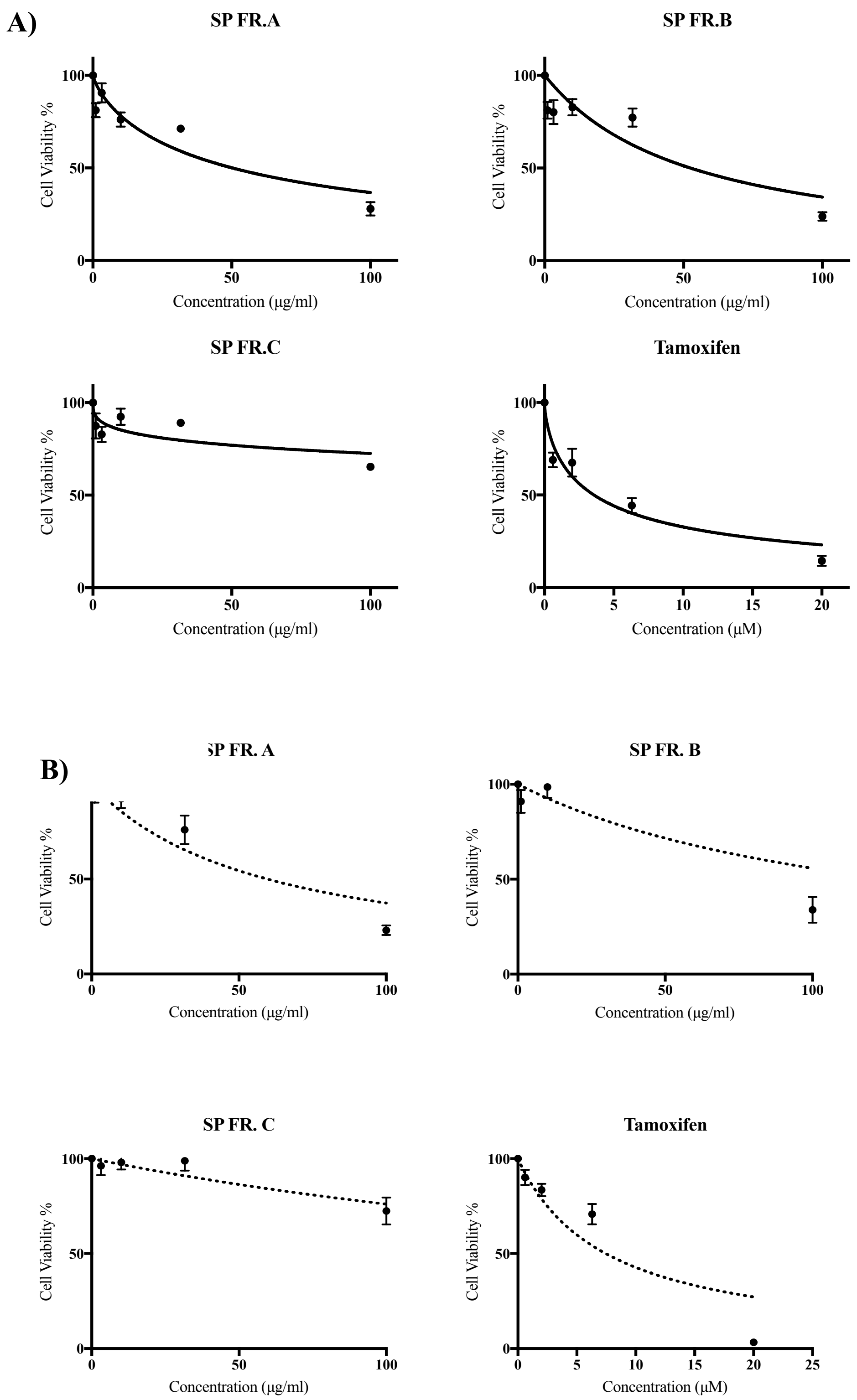

Figure S22. Cytotoxic effects of the SP LH-20 column fractions of the CA EtOAc extract on the MCF-7 (A) and MCF-10A (B) cell lines. Cells were treated with column fractions of *Citrus aurantium* ethyl acetate extract in 48 h and % cell viabilities were measured.

A)

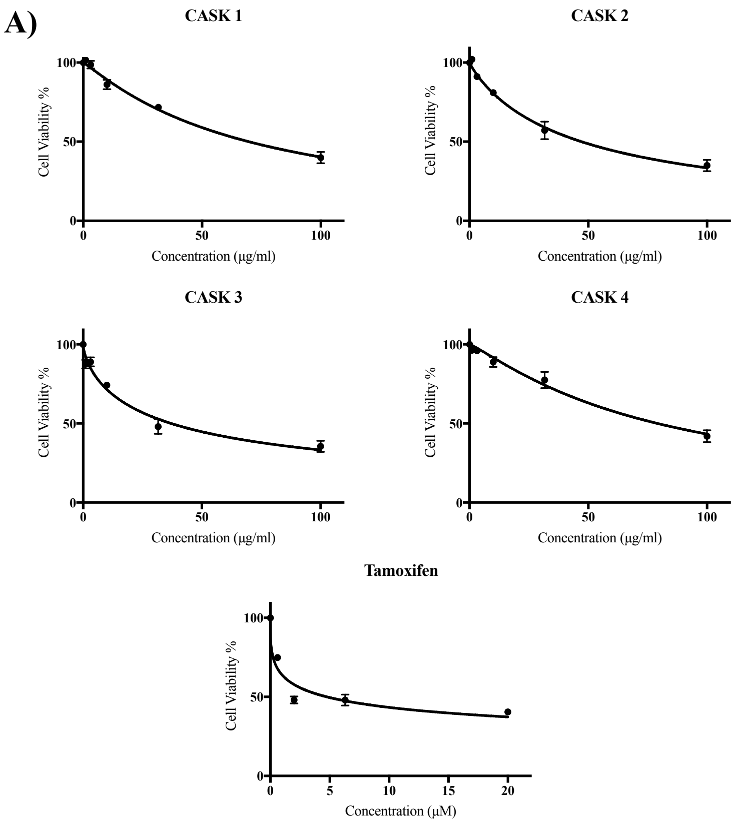

B)

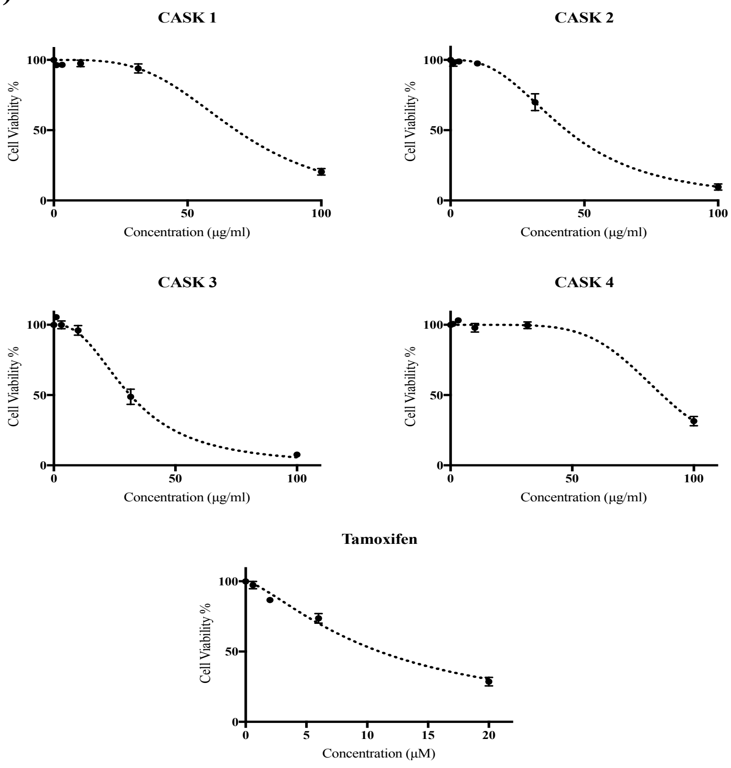

Figure S23. Cytotoxic effects of the silica gel column fractions (sub-fractions) on the MCF-7 (A) and MCF-10A (B) cell lines. Cells were treated with CASK1, CASK2, CASK3, CASK4 subfractions in 48 h and % cell viabilities were measured.

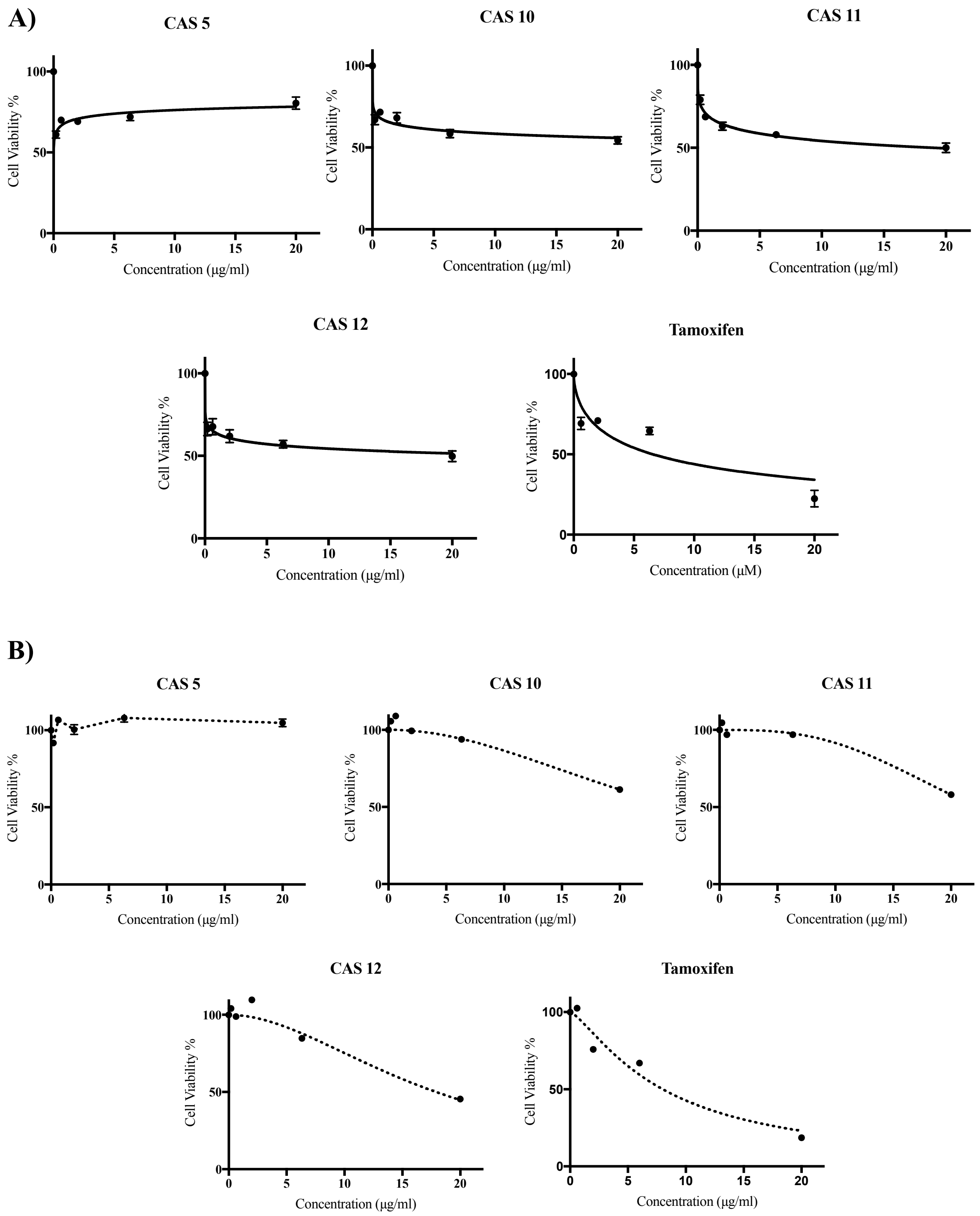

Figure S24. Cytotoxic effects of compounds isolated from CASK 3 on the MCF-7 (A) and MCF-10A (B) cell lines. Cells were treated with isolated compounds (CAS5, CAS10, CAS11, CAS12) in 48 h and % cell viabilities were measured.
